# Dung-beetles (Coleoptera, Scarabaeidae, Aphodiinae, Scarabaeinae) feeding on faeces of steppe marmots *Marmotabobak* (Rodentia, Sciuridae) in Middle Volga territory

**DOI:** 10.3897/BDJ.12.e125090

**Published:** 2024-06-18

**Authors:** Lilia A. Akhmetova, Andrei S. Kurochkin, Andrey V. Frolov

**Affiliations:** 1 Zoological Institute RAS, Saint-Petersburg, Russia Zoological Institute RAS Saint-Petersburg Russia; 2 Samara National Research University, Department of Ecology, Botany and Nature Protection, Samara, Russia Samara National Research University, Department of Ecology, Botany and Nature Protection Samara Russia

**Keywords:** steppe marmot, ground squirrels, keystone species, scarab beetles, dung-beetles, coprophagy

## Abstract

**Background:**

In open terrestrial biomes of Holarctic realm, ground squirrels are recognised as keystone species inhabiting steppes. They shape the plant species composition and diversity and support a fauna of species associated with their burrows. Ground squirrels and associated dung-beetles are important elements of the steppe food webs, yet the trophic associations between species are still poorly studied.

**New information:**

The area in the northern outskirts of Obshchy Syrt plateau, on the border of Samara and Orenburg Provinces of Russia was surveyed and scarab beetles (Scarabaeidae) feeding on steppe marmot (*Marmotabobak* (Müller, 1776)) faeces were collected from six localities. Twenty eight species of two subfamilies – Aphodiinae and Scarabaeinae, - were identified with the majority of species belonging the genus *Aphodius* Hellwig, 1798. Seven species are recorded as consumers of marmot faeces for the first time. Only two nidicolous specialist species were found which suggests that the studied population of steppe marmots is as result of the recent secondary colonisation and not all the associated scarab beetle faunas were re-established.

## Introduction

The concept of keystone species has become established in ecology and conservation research, although the term “keystone species” has varied meanings in literature (Mills et al. 1993). In the most general meaning, keystone species are those which presence is crucial for maintaining the diversity of their ecological communities and which importance is disproportionally large relative to their abundance (Paine 1969). In the open terrestrial biomes of the Holarctic realm, ground squirrels of the tribe Marmotini Pocock, 1923, are commonly recognised as keystone species inhabiting steppes (Lindtner et al. 2020, Lindtner et al. 2018). Large ground squirrels of the genus *Marmota* Blumenbach, 1779, comprise 15 species. The species of this genus were a subject of long-lasting research because of their importance and, also, as the main carriers of plague in some areas.

A nidicolous fauna associated with burrows of ground squirrels was studied by a number of authors. The scarab beetle fauna inhabiting marmot burrows in Russia is dealt with in a few studies (Egorov 1997, Zinchenko and Nemkov 1998, Zinchenko 2003). Two new species of the genus *Aphodius*, *A.isajevi* Kabakov, 1994 and *A.exilimanus* Kabakov, 1994, were described in the past decades from marmot burrows (Kabakov 1994) and they are considered specialist nidicolous. However, these studies were focused on the documentation of the fauna found in the borrows and not on the trophic associations of beetles and marmots.

Recently, we conducted field research in Bol’shechernigovskij District, Samara Province and the Pervomaiskij District, Orenburg Province, European Russia. The area is inhabited by steppe marmot (*Marmotabobak* (Müller, 1776)). As a result of the survey, a large number of dung-beetle species were registered feeding on the steppe marmot excrements. The goal of the present contribution is to report the registered trophic associations of scarab beetles with steppe marmots and discuss, based on the associated beetles fauna, the possibility of native versus introduced origin of this marmot population.

## Materials and methods

### Collecting localities

**Figure 1.**
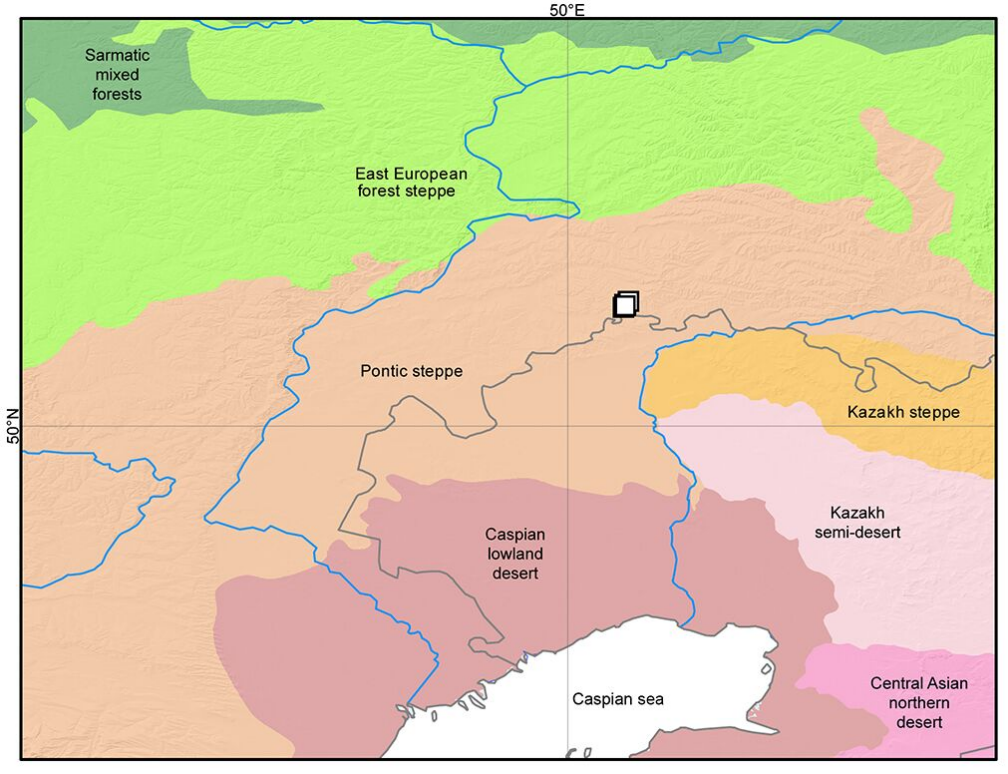
Collecting region.

**Figure 2.**
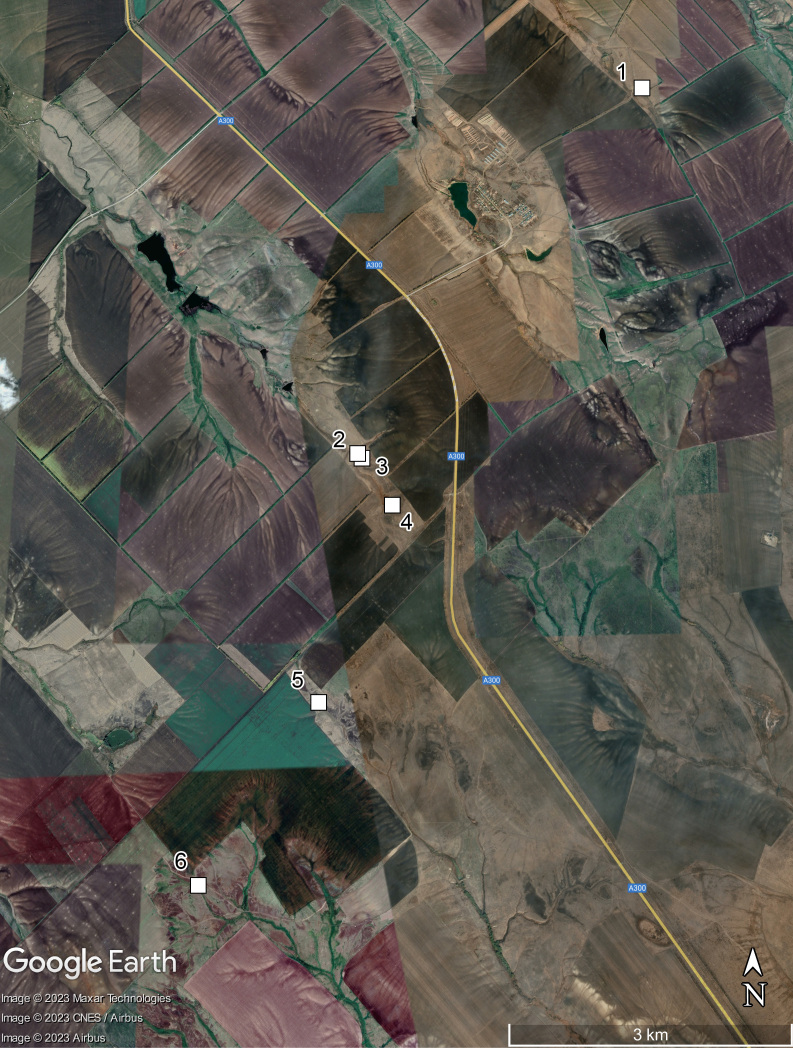
Collecting localities.

**Figure 3.**
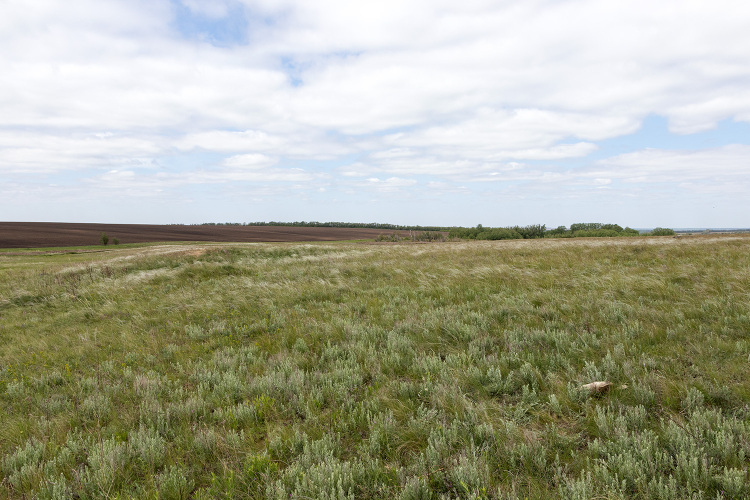
Locality 1. Bol’shechernigovskij Distr., 2.44 km SSW of Polyanskij Vill., gully Barsuchikha, forb-fescue-feather grass steppe.

**Figure 4.**
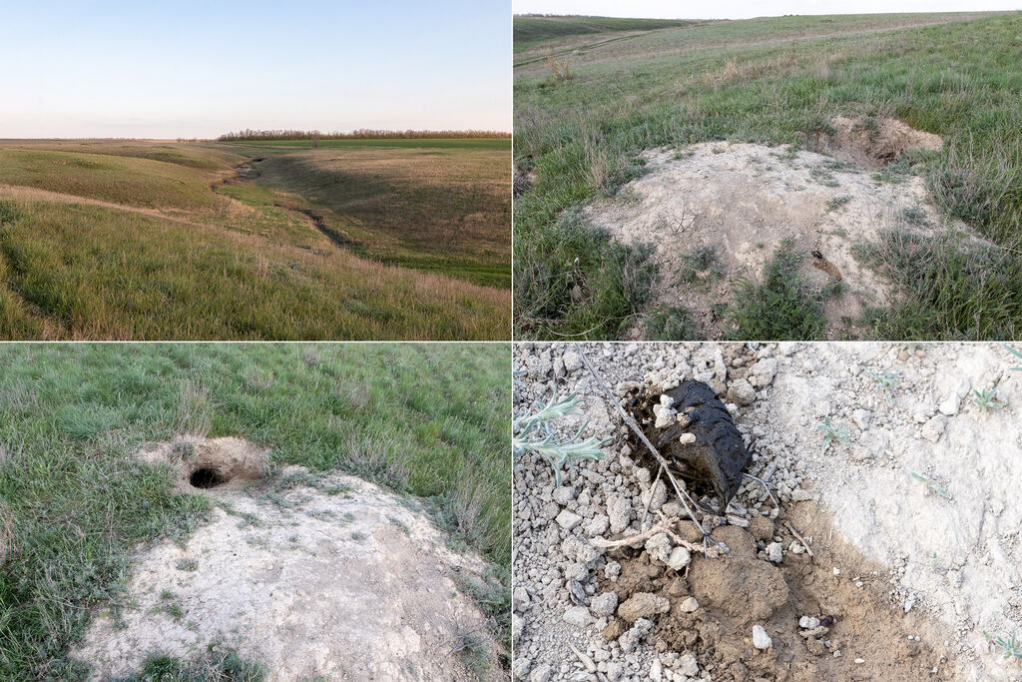
Locality 2. Bol’shechernigovskij Distr., 3.53 km SSW of Polyanskij Vill., gully slope, forb-grass steppe.

**Figure 5.**
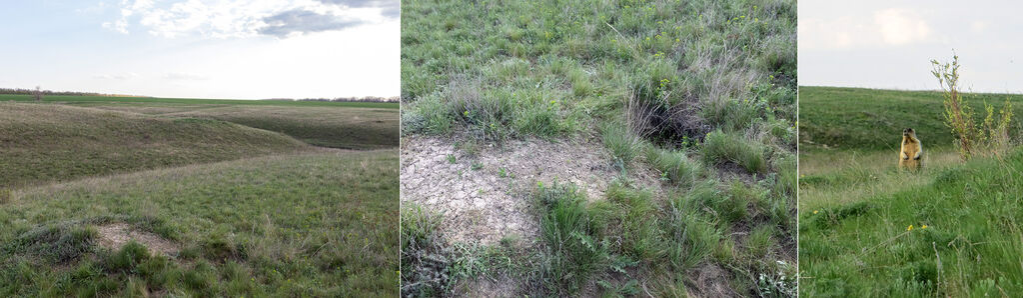
Locality 3. Bol’shechernigovskij Distr., 3.54 km SSW of Polyanskij Vill., gully slope, forb-grass steppe.

**Figure 6.**
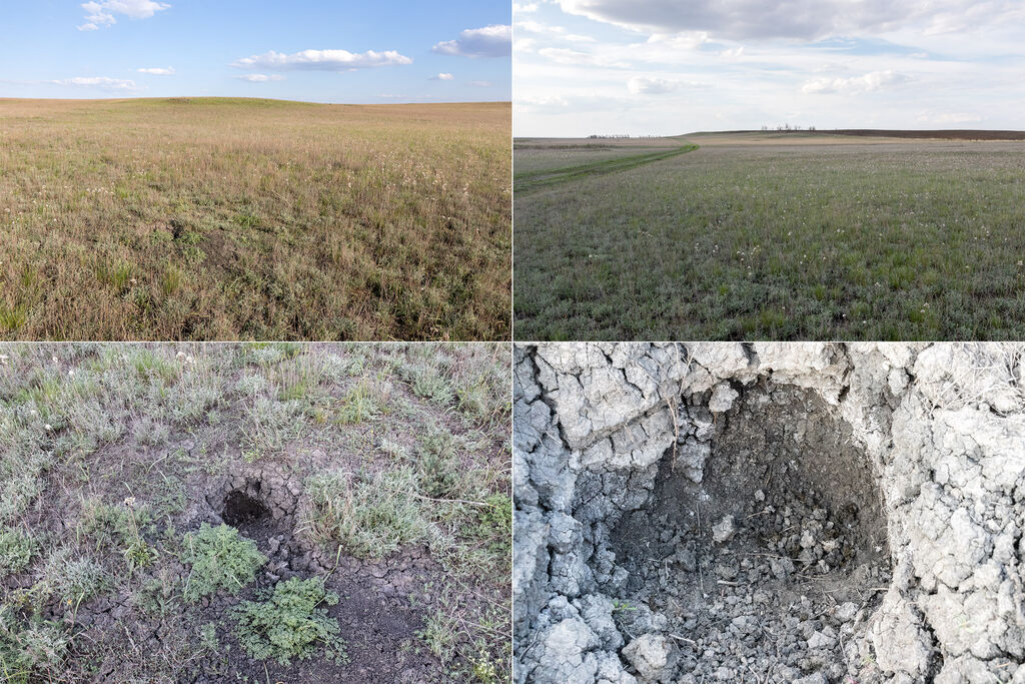
Locality 4. Bol’shechernigovskij Distr., 3.9 km SSW of Polyanskij Vill., gully slope, forb-grass steppe.

**Figure 7.**
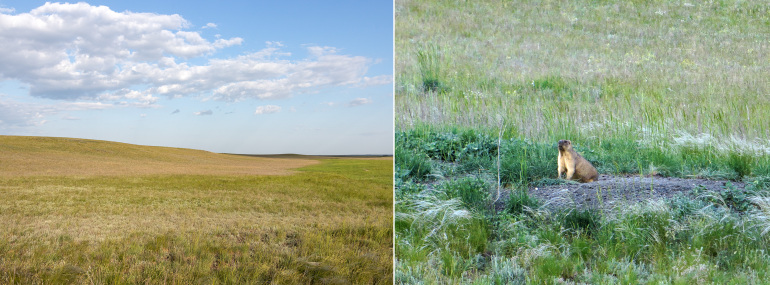
Locality 5. Pervomaiskij District, 6.28 km SSW of Polyanskij Vill., urochishche Pal’govo, forb-fescue steppe.

The collecting area is situated some 520 km north of the Caspian Sea coast, in the Pontic steppe ecozone (Fig. 1). The six sampling localities are situated mostly between crop fields with outermost localities separated by about 10 km (Fig. 2). The characterisation of the localities is given in Table 1 and habitat illustrations are given in Figs 3, 4, 5, 6, 7.

### Material collecting and deposition and data presentation

Beetles were collected in latrines near marmot burrow entrances. The beetles were picked up from faeces and upper soil layer beneath faeces with a forceps and placed in tubes with ethanol. Individuals, arriving at faeces during survey, were also collected. All collected material is housed the the Zoological Institute RAS, Saint-Petersburg, Russia (ZIN).

Classification, distribution and biology of Aphodiinae species follow Akhmetova and Frolov (2014) and that of Scarabaenae follow Kabakov (2006), Bezděk (2016a), Bezděk (2016b), Ziani and Bezděk (2016). Locality maps were generated with ArcGIS 10.6 (ESRI Ltd., USA) and Google Earth (Google, USA) software. For publishing trophic associations via nanopublications, Nanodash platform (https://nanodash.petapico.org/) was used.

## Taxon treatments

### Aphodius (Plagiogonus) arenarius

Olivier, 1789

3E38AA1B-E367-578E-A9A1-60D01E070FF4

#### Materials

- **Type status:** Other material. **Occurrence:** recordedBy: A.S. Kurochkin; individualCount: 1; lifeStage: adult; occurrenceID: FC307EB8-3A06-5975-8EBF-5D72ABB1C638; **Taxon:** scientificName: Aphodiusarenarius; kingdom: Animalia; phylum: Arthropoda; class: Insecta ; order: Coleoptera; family: Scarabaeidae; genus: Aphodius; taxonRank: species; **Location:** country: Russia; stateProvince: Samara; locality: Bol’shechernigovskij distr., 3.9 km SSW of Polyanskij vill., gully bottom, forb-grass steppe.; decimalLatitude: 51.85; decimalLongitude: 50.89; georeferenceProtocol: GPS; **Identification:** identifiedBy: L.A.Akhmetova; dateIdentified: 2023; **Event:** samplingProtocol: From feces of Marmotabobak, in toilet burrow; eventDate: 2023-04-28; **Record Level:** collectionID: urn:lsid:biocol.org:col:34969; institutionCode: ZIN; collectionCode: Coleoptera; basisOfRecord: PreservedSpecimen

#### Distribution

This species is widely distributed in Europe, occurs also in the Caucasus, the Transcaucasus, Turkey, Kazakhstan and Turkmenistan. In Russia, it is known from the steppe zone from Kursk Prov. to West Siberia.

#### Biology

In Russia, the species occurs in rodent burrows (mostly of marmots and sousliks). In some localities, beetles can be found in large numbers near burrow entrances in May-June (Isajev 1995). However, in the UK, this species was collected under vegetable debris and sheep, cow and horse dung (Jessop 1986). It was also collected from rabbit latrines (Jason Mate, pers. comm.) Probably, in west Europe, in the areas without Marmotini rodents, this species develop in burrows of other rodents or rabbits, but its life cycle needs further research.

### Aphodius (Phalacronothus) biguttatus

Germar, 1824

5D3A3B18-4202-5926-A641-703A9621AEC3

#### Materials

- **Type status:** Other material. **Occurrence:** recordedBy: A.S. Kurochkin; individualCount: 3; lifeStage: adult; occurrenceID: 4E011CDD-D0D7-577F-8A1F-87E6B16A6258; **Taxon:** scientificName: Aphodiusbiguttatus; kingdom: Animalia; phylum: Arthropoda; class: Insecta ; order: Coleoptera; family: Scarabaeidae; genus: Aphodius; taxonRank: species; **Location:** country: Russia; stateProvince: Orenburg; locality: Pervomaiskij Distr., 6.28 km SSW of Polyanskij vill., urochishche Pal’govo, gully bottom, forb-fescue steppe.; decimalLatitude: 51.83; decimalLongitude: 50.87; georeferenceProtocol: GPS; **Identification:** identifiedBy: L.A.Akhmetova; dateIdentified: 2023; **Event:** samplingProtocol: From feces of Marmotabobak, near burrow entrance; eventDate: 2023-05-18; **Record Level:** collectionID: urn:lsid:biocol.org:col:34969; institutionCode: ZIN; collectionCode: Coleoptera
- **Type status:** Other material. **Occurrence:** recordedBy: A.S. Kurochkin; individualCount: 8; lifeStage: adult; occurrenceID: 0EE7F74C-1385-5364-A563-53EFEE4417D2; **Taxon:** scientificName: Aphodiusbiguttatus; kingdom: Animalia; phylum: Arthropoda; class: Insecta ; order: Coleoptera; family: Scarabaeidae; genus: Aphodius; taxonRank: species; **Location:** country: Russia; stateProvince: Samara; locality: Bol’shechernigovskij distr., 3.9 km SSW of Polyanskij vill., gully bottom, forb-grass steppe.; decimalLatitude: 51.85; decimalLongitude: 50.89; georeferenceProtocol: GPS; **Identification:** identifiedBy: L.A.Akhmetova; dateIdentified: 2023; **Event:** samplingProtocol: From feces of Marmotabobak, in toilet burrow; eventDate: 2023-04-28; **Record Level:** collectionID: urn:lsid:biocol.org:col:34969; institutionCode: ZIN; collectionCode: Coleoptera
- **Type status:** Other material. **Occurrence:** recordedBy: A.S. Kurochkin; individualCount: 2; lifeStage: adult; occurrenceID: DF289CBB-5353-5AA3-953D-46294F591006; **Taxon:** scientificName: Aphodiusbiguttatus; kingdom: Animalia; phylum: Arthropoda; class: Insecta ; order: Coleoptera; family: Scarabaeidae; genus: Aphodius; taxonRank: species; **Location:** country: Russia; stateProvince: Samara; locality: Bol’shechernigovskij distr., 2.44 km SSW of Polyanskij vill., gully Barsuchikha, forb-fescue-feather grass steppe; decimalLatitude: 51.9; decimalLongitude: 50.93; georeferenceProtocol: GPS; **Identification:** identifiedBy: L.A.Akhmetova; dateIdentified: 2023; **Event:** samplingProtocol: From feces of Marmotabobak, in toilet burrow; eventDate: 2023-05-19; **Record Level:** collectionID: urn:lsid:biocol.org:col:34969; institutionCode: ZIN; collectionCode: Coleoptera
- **Type status:** Other material. **Occurrence:** recordedBy: A.S. Kurochkin; individualCount: 4; lifeStage: adult; occurrenceID: 1AF7BD20-CCAA-5E5F-AA29-EFFC848823E6; **Taxon:** scientificName: Aphodiusbiguttatus; kingdom: Animalia; phylum: Arthropoda; class: Insecta ; order: Coleoptera; family: Scarabaeidae; genus: Aphodius; taxonRank: species; **Location:** country: Russia; stateProvince: Samara; locality: Bol’shechernigovskij distr., 3.54 km SSW of Polyanskij vill., gully bottom, forb-grass steppe.; decimalLatitude: 51.86; decimalLongitude: 50.88; georeferenceProtocol: GPS; **Identification:** identifiedBy: L.A.Akhmetova; dateIdentified: 2023; **Event:** samplingProtocol: From feces of Marmotabobak, near burrow entrance; eventDate: 2023-04-28; **Record Level:** collectionID: urn:lsid:biocol.org:col:34969; institutionCode: ZIN; collectionCode: Coleoptera
- **Type status:** Other material. **Occurrence:** recordedBy: A.S. Kurochkin; individualCount: 1; lifeStage: adult; occurrenceID: AB88C9D8-F4AC-5D8A-9BFE-D8C2F07668E5; **Taxon:** scientificName: Aphodiusbiguttatus; kingdom: Animalia; phylum: Arthropoda; class: Insecta ; order: Coleoptera; family: Scarabaeidae; genus: Aphodius; taxonRank: species; **Location:** country: Russia; stateProvince: Samara; locality: Bol’shechernigovskij distr., 3.54 km SSW of Polyanskij vill., gully bottom, forb-grass steppe.; decimalLatitude: 51.86; decimalLongitude: 50.88; georeferenceProtocol: GPS; **Identification:** identifiedBy: L.A.Akhmetova; dateIdentified: 2023; **Event:** samplingProtocol: From feces of Marmotabobak, near burrow entrance; eventDate: 2023-04-28; **Record Level:** collectionID: urn:lsid:biocol.org:col:34969; institutionCode: ZIN; collectionCode: Coleoptera

#### Distribution

The species is distributed in central, south and eastern Europe, the Transcaucasus, Asia Minor, Kazakhstan and Turkmenistan. In Russia, it is known from Ulyanovsk and Novosibirsk Provinces, Volgograd, Rostov, Orenburg Provinces and Krasnoyarsk Terr.

#### Biology

The species occurs in open biotopes, in marmot holes and in the dung of domestic animals.

### Aphodius (Acrossus) bimaculatus

(Laxmann, 1770)

8D700915-FCA9-56D5-A4D7-36B6F99056C0

#### Materials

- **Type status:** Other material. **Occurrence:** recordedBy: A.S. Kurochkin; individualCount: 2; lifeStage: adult; occurrenceID: C9ECDDBE-9CBC-5D5D-8D25-AD6C3BAE7150; **Taxon:** scientificName: Aphodiusbimaculatus; kingdom: Animalia; phylum: Arthropoda; class: Insecta ; order: Coleoptera; family: Scarabaeidae; genus: Aphodius; taxonRank: species; **Location:** country: Russia; stateProvince: Orenburg; locality: Pervomaiskij Distr., 6.28 km SSW of Polyanskij vill., urochishche Pal’govo, gully bottom, forb-fescue steppe.; decimalLatitude: 51.83; decimalLongitude: 50.87; georeferenceProtocol: GPS; **Identification:** identifiedBy: L.A.Akhmetova; dateIdentified: 2023; **Event:** samplingProtocol: From feces of Marmotabobak, near burrow entrance; eventDate: 2023-05-18; **Record Level:** collectionID: urn:lsid:biocol.org:col:34969; institutionCode: ZIN; collectionCode: Coleoptera

#### Distribution

The species occurs in central and eastern Europe, north and eastern Kazakhstan, Kyrgyzstan. In Russia, it is known from the European part and West Siberia (up to Krasnoyarsk in the north-east).

#### Biology

The beetles and larvae feed on horse dung.

### Aphodius (Phalacronothus) citellorum

Semenov et Medvedev, 1928

B73C9B87-14F5-5A99-89B3-D45A28198477

#### Materials

- **Type status:** Other material. **Occurrence:** recordedBy: A.S. Kurochkin; individualCount: 6; lifeStage: adult; occurrenceID: C2361360-713B-5DC6-BC0A-9309FDB7334F; **Taxon:** scientificName: Aphodiuscitellorum; kingdom: Animalia; phylum: Arthropoda; class: Insecta ; order: Coleoptera; family: Scarabaeidae; genus: Aphodius; taxonRank: species; **Location:** country: Russia; stateProvince: Samara; locality: Bol’shechernigovskij distr., 3.9 km SSW of Polyanskij vill., gully bottom, forb-grass steppe.; decimalLatitude: 51.85; decimalLongitude: 50.89; georeferenceProtocol: GPS; **Identification:** identifiedBy: L.A.Akhmetova; dateIdentified: 2023; **Event:** samplingProtocol: From feces of Marmotabobak, in toilet burrow; eventDate: 2023-04-28; **Record Level:** collectionID: urn:lsid:biocol.org:col:34969; institutionCode: ZIN; collectionCode: Coleoptera

#### Distribution

The species is distributed in the steppe zone of central and eastern Europe and Kazakhstan.

#### Biology

A nidicolous steppe species occurring in gopher and marmot holes.

### Aphodius (Euorodalus) coenosus

(Panzer, 1798)

F045935E-5BC7-54DC-8D31-E826EDB69D02

#### Materials

- **Type status:** Other material. **Occurrence:** recordedBy: A.S. Kurochkin; individualCount: 1; lifeStage: adult; occurrenceID: 08E1A3B1-DD29-52C3-BDA8-4CCBC65921C9; **Taxon:** scientificName: Aphodiuscoenosus; kingdom: Animalia; phylum: Arthropoda; class: Insecta ; order: Coleoptera; family: Scarabaeidae; genus: Aphodius; taxonRank: species; **Location:** country: Russia; stateProvince: Orenburg; locality: Pervomaiskij Distr., 6.28 km SSW of Polyanskij vill., urochishche Pal’govo, gully bottom, forb-fescue steppe.; decimalLatitude: 51.83; decimalLongitude: 50.87; georeferenceProtocol: GPS; **Identification:** identifiedBy: L.A.Akhmetova; dateIdentified: 2023; **Event:** samplingProtocol: From feces of Marmotabobak, near burrow entrance; eventDate: 2023-05-18; **Record Level:** collectionID: urn:lsid:biocol.org:col:34969; institutionCode: ZIN; collectionCode: Coleoptera
- **Type status:** Other material. **Occurrence:** recordedBy: A.S. Kurochkin; individualCount: 1; lifeStage: adult; occurrenceID: F383FB0B-9502-5C14-A07F-5663DCB4F9F1; **Taxon:** scientificName: Aphodiuscoenosus; kingdom: Animalia; phylum: Arthropoda; class: Insecta ; order: Coleoptera; family: Scarabaeidae; genus: Aphodius; taxonRank: species; **Location:** country: Russia; stateProvince: Samara; locality: Bol’shechernigovskij distr., 3.9 km SSW of Polyanskij vill., gully bottom, forb-grass steppe.; decimalLatitude: 51.85; decimalLongitude: 50.89; georeferenceProtocol: GPS; **Identification:** identifiedBy: L.A.Akhmetova; dateIdentified: 2023; **Event:** samplingProtocol: From feces of Marmotabobak, in toilet burrow; eventDate: 2023-04-28; **Record Level:** collectionID: urn:lsid:biocol.org:col:34969; institutionCode: ZIN; collectionCode: Coleoptera
- **Type status:** Other material. **Occurrence:** recordedBy: A.S. Kurochkin; individualCount: 2; lifeStage: adult; occurrenceID: 409FA26D-688C-5576-A9FA-EC78B9379AF6; **Taxon:** scientificName: Aphodiuscoenosus; kingdom: Animalia; phylum: Arthropoda; class: Insecta ; order: Coleoptera; family: Scarabaeidae; genus: Aphodius; taxonRank: species; **Location:** country: Russia; stateProvince: Samara; locality: Bol’shechernigovskij distr., 2.44 km SSW of Polyanskij vill., gully Barsuchikha, forb-fescue-feather grass steppe; decimalLatitude: 51.9; decimalLongitude: 50.93; georeferenceProtocol: GPS; **Identification:** identifiedBy: L.A.Akhmetova; dateIdentified: 2023; **Event:** samplingProtocol: From feces of Marmotabobak, in toilet burrow; eventDate: 2023-05-19; **Record Level:** collectionID: urn:lsid:biocol.org:col:34969; institutionCode: ZIN; collectionCode: Coleoptera
- **Type status:** Other material. **Occurrence:** recordedBy: A.S. Kurochkin; individualCount: 1; lifeStage: adult; occurrenceID: 3F044CFE-5745-5150-89CF-632D1ABF854C; **Taxon:** scientificName: Aphodiuscoenosus; kingdom: Animalia; phylum: Arthropoda; class: Insecta ; order: Coleoptera; family: Scarabaeidae; genus: Aphodius; taxonRank: species; **Location:** country: Russia; stateProvince: Samara; locality: Bol’shechernigovskij distr., 3.54 km SSW of Polyanskij vill., gully bottom, forb-grass steppe.; decimalLatitude: 51.86; decimalLongitude: 50.88; georeferenceProtocol: GPS; **Identification:** identifiedBy: L.A.Akhmetova; dateIdentified: 2023; **Event:** samplingProtocol: From feces of Marmotabobak, near burrow entrance; eventDate: 2023-04-28; **Record Level:** collectionID: urn:lsid:biocol.org:col:34969; institutionCode: ZIN; collectionCode: Coleoptera

#### Distribution

The distribution range of this species includes almost the whole of Europe (except for the extreme north), the Transcaucasus, Asia Minor, west Kazakhstan and Tajikistan. In Russia, the species is known from Leningrad, Nizhny Novgorod, Voronezh, Saratov, Volgograd and Astrakhan Provinces.

#### Biology

A coprophagous species occurring in the dung of cows, horses and wild ungulates. The species occurs in different biotopes: in eastern Europe, in forests; Isajev (1995) recorded it from steppes; we collected it on sandy soils on the Akhtuba riverside in Dosang environs (Astrakhan Prov.).

### Aphodius (Chilothorax) distinctus

(Müller, 1776)

FA45715B-C74E-5759-8C65-E3596AAD656E

#### Materials

- **Type status:** Other material. **Occurrence:** recordedBy: A.S. Kurochkin; individualCount: 20; lifeStage: adult; occurrenceID: 59DD7651-D4BC-5F25-9101-7A7397105D33; **Taxon:** scientificName: Aphodiusdistinctus; kingdom: Animalia; phylum: Arthropoda; class: Insecta ; order: Coleoptera; family: Scarabaeidae; genus: Aphodius; taxonRank: species; **Location:** country: Russia; stateProvince: Orenburg; locality: Pervomaiskij Distr., 6.28 km SSW of Polyanskij vill., urochishche Pal’govo, gully bottom, forb-fescue steppe.; decimalLatitude: 51.83; decimalLongitude: 50.87; georeferenceProtocol: GPS; **Identification:** identifiedBy: L.A.Akhmetova; dateIdentified: 2023; **Event:** samplingProtocol: From feces of Marmotabobak, near burrow entrance; eventDate: 2023-05-18; **Record Level:** collectionID: urn:lsid:biocol.org:col:34969; institutionCode: ZIN; collectionCode: Coleoptera
- **Type status:** Other material. **Occurrence:** recordedBy: A.S. Kurochkin; individualCount: 2; lifeStage: adult; occurrenceID: CFB8A81B-F231-5DD5-832D-A6D7CB745766; **Taxon:** scientificName: Aphodiusdistinctus; kingdom: Animalia; phylum: Arthropoda; class: Insecta ; order: Coleoptera; family: Scarabaeidae; genus: Aphodius; taxonRank: species; **Location:** country: Russia; stateProvince: Orenburg; locality: Pervomaiskij Distr., 7.32 km SSW of Koshkin vill., urochishche Pal’govo, gully bottom, forb-wormwood-grass steppe.; decimalLatitude: 51.82; decimalLongitude: 50.86; georeferenceProtocol: GPS; **Identification:** identifiedBy: L.A.Akhmetova; dateIdentified: 2023; **Event:** samplingProtocol: From feces of Marmotabobak, near burrow entrance; eventDate: 2023-05-19; **Record Level:** collectionID: urn:lsid:biocol.org:col:34969; institutionCode: ZIN; collectionCode: Coleoptera
- **Type status:** Other material. **Occurrence:** recordedBy: A.S. Kurochkin; individualCount: 19; lifeStage: adult; occurrenceID: 27D76291-9718-5512-B57E-CFF64E13E66F; **Taxon:** scientificName: Aphodiusdistinctus; kingdom: Animalia; phylum: Arthropoda; class: Insecta ; order: Coleoptera; family: Scarabaeidae; genus: Aphodius; taxonRank: species; **Location:** country: Russia; stateProvince: Samara; locality: Bol’shechernigovskij distr., 3.9 km SSW of Polyanskij vill., gully bottom, forb-grass steppe.; decimalLatitude: 51.85; decimalLongitude: 50.89; georeferenceProtocol: GPS; **Identification:** identifiedBy: L.A.Akhmetova; dateIdentified: 2023; **Event:** samplingProtocol: From feces of Marmotabobak, in toilet burrow; eventDate: 2023-04-28; **Record Level:** collectionID: urn:lsid:biocol.org:col:34969; institutionCode: ZIN; collectionCode: Coleoptera
- **Type status:** Other material. **Occurrence:** recordedBy: A.S. Kurochkin; individualCount: 7; lifeStage: adult; occurrenceID: 8058494E-969C-53C0-9E53-210B11088D8B; **Taxon:** scientificName: Aphodiusdistinctus; kingdom: Animalia; phylum: Arthropoda; class: Insecta ; order: Coleoptera; family: Scarabaeidae; genus: Aphodius; taxonRank: species; **Location:** country: Russia; stateProvince: Samara; locality: Bol’shechernigovskij distr., 2.44 km SSW of Polyanskij vill., gully Barsuchikha, forb-fescue-feather grass steppe; decimalLatitude: 51.9; decimalLongitude: 50.93; georeferenceProtocol: GPS; **Identification:** identifiedBy: L.A.Akhmetova; dateIdentified: 2023; **Event:** samplingProtocol: From feces of Marmotabobak, in toilet burrow; eventDate: 2023-05-19; **Record Level:** collectionID: urn:lsid:biocol.org:col:34969; institutionCode: ZIN; collectionCode: Coleoptera
- **Type status:** Other material. **Occurrence:** recordedBy: A.S. Kurochkin; individualCount: 10; lifeStage: adult; occurrenceID: 3D99508F-1B50-54B9-81BF-3B0677F6E224; **Taxon:** scientificName: Aphodiusdistinctus; kingdom: Animalia; phylum: Arthropoda; class: Insecta ; order: Coleoptera; family: Scarabaeidae; genus: Aphodius; taxonRank: species; **Location:** country: Russia; stateProvince: Samara; locality: Bol’shechernigovskij distr., 3.54 km SSW of Polyanskij vill., gully bottom, forb-grass steppe.; decimalLatitude: 51.86; decimalLongitude: 50.88; georeferenceProtocol: GPS; **Identification:** identifiedBy: L.A.Akhmetova; dateIdentified: 2023; **Event:** samplingProtocol: From feces of Marmotabobak, near burrow entrance; eventDate: 2023-04-28; **Record Level:** collectionID: urn:lsid:biocol.org:col:34969; institutionCode: ZIN; collectionCode: Coleoptera
- **Type status:** Other material. **Occurrence:** recordedBy: A.S. Kurochkin; individualCount: 45; lifeStage: adult; occurrenceID: 6D0FBCCF-B305-5618-9368-C02AA7075BF5; **Taxon:** scientificName: Aphodiusdistinctus; kingdom: Animalia; phylum: Arthropoda; class: Insecta ; order: Coleoptera; family: Scarabaeidae; genus: Aphodius; taxonRank: species; **Location:** country: Russia; stateProvince: Samara; locality: Bol’shechernigovskij distr., 3.54 km SSW of Polyanskij vill., gully bottom, forb-grass steppe.; decimalLatitude: 51.86; decimalLongitude: 50.88; georeferenceProtocol: GPS; **Identification:** identifiedBy: L.A.Akhmetova; dateIdentified: 2023; **Event:** samplingProtocol: From feces of Marmotabobak, near burrow entrance; eventDate: 2023-04-28; **Record Level:** collectionID: urn:lsid:biocol.org:col:34969; institutionCode: ZIN; collectionCode: Coleoptera
- **Type status:** Other material. **Occurrence:** recordedBy: A.S. Kurochkin; individualCount: 2; lifeStage: adult; occurrenceID: 84FEE59C-19AB-5E81-9A46-4033DC9E3D18; **Taxon:** scientificName: Aphodiusdistinctus; kingdom: Animalia; phylum: Arthropoda; class: Insecta ; order: Coleoptera; family: Scarabaeidae; genus: Aphodius; taxonRank: species; **Location:** country: Russia; stateProvince: Samara; locality: Bol’shechernigovskij distr., 3.53 km SSW of Polyanskij vill., gully bottom, forb-grass steppe.; decimalLatitude: 51.86; decimalLongitude: 50.88; georeferenceProtocol: GPS; **Identification:** identifiedBy: L.A.Akhmetova; dateIdentified: 2023; **Event:** samplingProtocol: From feces of Marmotabobak, near burrow entrance; eventDate: 2023-05-19; **Record Level:** collectionID: urn:lsid:biocol.org:col:34969; institutionCode: ZIN; collectionCode: Coleoptera

#### Distribution

The distribution range of this species includes the whole of Europe (except for the extreme north), North Africa, the Transcaucasus, Asia Minor, Kazakhstan, Middle Asia, Mongolia. It was imported to North America. In Russia, it occurs throughout the country in the European part, reaching Baikal Region in the east.

#### Biology

The species occurs in cow and horse dung, in marmot holes and also is attracted to light. In Russia, the beetles are active from March to October. It is a common, locally abundant species.

### Aphodius (Colobopterus) erraticus

(Linnaeus, 1758)

24D8EBE4-55DB-57A1-9710-C7543095A699

#### Materials

- **Type status:** Other material. **Occurrence:** recordedBy: A.S. Kurochkin; individualCount: 2; lifeStage: adult; occurrenceID: FF3FC2BA-042F-539C-A636-EBAC31DD9824; **Taxon:** scientificName: Aphodiuserraticus; kingdom: Animalia; phylum: Arthropoda; class: Insecta ; order: Coleoptera; family: Scarabaeidae; genus: Aphodius; taxonRank: species; **Location:** country: Russia; stateProvince: Samara; locality: Bol’shechernigovskij distr., 3.9 km SSW of Polyanskij vill., gully bottom, forb-grass steppe.; decimalLatitude: 51.85; decimalLongitude: 50.89; georeferenceProtocol: GPS; **Identification:** identifiedBy: L.A.Akhmetova; dateIdentified: 2023; **Event:** samplingProtocol: From feces of Marmotabobak, in toilet burrow; eventDate: 2023-04-28; **Record Level:** collectionID: urn:lsid:biocol.org:col:34969; institutionCode: ZIN; collectionCode: Coleoptera
- **Type status:** Other material. **Occurrence:** recordedBy: A.S. Kurochkin; individualCount: 1; lifeStage: adult; occurrenceID: 4CDE1063-CA63-5478-9670-A5C1FA9C474A; **Taxon:** scientificName: Aphodiuserraticus; kingdom: Animalia; phylum: Arthropoda; class: Insecta ; order: Coleoptera; family: Scarabaeidae; genus: Aphodius; taxonRank: species; **Location:** country: Russia; stateProvince: Samara; locality: Bol’shechernigovskij distr., 3.54 km SSW of Polyanskij vill., gully bottom, forb-grass steppe.; decimalLatitude: 51.86; decimalLongitude: 50.88; georeferenceProtocol: GPS; **Identification:** identifiedBy: L.A.Akhmetova; dateIdentified: 2023; **Event:** samplingProtocol: From feces of Marmotabobak, near burrow entrance; eventDate: 2023-04-28; **Record Level:** collectionID: urn:lsid:biocol.org:col:34969; institutionCode: ZIN; collectionCode: Coleoptera

#### Distribution

The species occurs in Europe (except for the extreme north), west, middle and Central Asia; it was imported to North America. In Russia, it is distributed throughout the country.

#### Biology

This eurybiont species is abundant throughout its range occurring mostly in open biotopes, in the dung of domestic and wild animals.

### Aphodius (Aphodius) fimetarius

(Linnaeus, 1758)

CC11689F-7307-53C2-8626-15B106FD4489

#### Materials

- **Type status:** Other material. **Occurrence:** recordedBy: A.S. Kurochkin; individualCount: 1; lifeStage: adult; occurrenceID: 161382BF-8AD4-504E-B4F0-EAEAA8D33415; **Taxon:** scientificName: Aphodiusfimetarius; kingdom: Animalia; phylum: Arthropoda; class: Insecta ; order: Coleoptera; family: Scarabaeidae; genus: Aphodius; taxonRank: species; **Location:** country: Russia; stateProvince: Samara; locality: Bol’shechernigovskij distr., 3.9 km SSW of Polyanskij vill., gully bottom, forb-grass steppe.; decimalLatitude: 51.85; decimalLongitude: 50.89; georeferenceProtocol: GPS; **Identification:** identifiedBy: L.A.Akhmetova; dateIdentified: 2023; **Event:** samplingProtocol: From feces of Marmotabobak, in toilet burrow; eventDate: 2023-04-28; **Record Level:** collectionID: urn:lsid:biocol.org:col:34969; institutionCode: ZIN; collectionCode: Coleoptera

#### Distribution

The species is widely distributed in Europe, North Africa, Kazakhstan, Middle and Central Asia, imported to North America and Australia. In Russia, it occurs throughout the country up to Eastern Siberia in the east.

#### Biology

In Russia, imagoes are active from April to October occurring in open biotopes, pastures, in cow, sheep and horse dung.

### Aphodius (Calamosternus) granarius

(Linnaeus,1767)

0ED41AEF-8DB8-5B49-A35A-0381192367B7

#### Materials

- **Type status:** Other material. **Occurrence:** recordedBy: A.S. Kurochkin; individualCount: 8; lifeStage: adult; occurrenceID: 5CA4435F-ECE7-5347-8AAB-A37B9453D49D; **Taxon:** scientificName: Aphodiusgranarius; kingdom: Animalia; phylum: Arthropoda; class: Insecta ; order: Coleoptera; family: Scarabaeidae; genus: Aphodius; taxonRank: species; **Location:** country: Russia; stateProvince: Orenburg; locality: Pervomaiskij Distr., 6.28 km SSW of Polyanskij vill., urochishche Pal’govo, gully bottom, forb-fescue steppe.; decimalLatitude: 51.83; decimalLongitude: 50.87; georeferenceProtocol: GPS; **Identification:** identifiedBy: L.A.Akhmetova; dateIdentified: 2023; **Event:** samplingProtocol: From feces of Marmotabobak, near burrow entrance; eventDate: 2023-05-18; **Record Level:** collectionID: urn:lsid:biocol.org:col:34969; institutionCode: ZIN; collectionCode: Coleoptera

#### Distribution

The species is distributed throughout Europe (except for the extreme north), west Asia, Kazakhstan and Middle Asia; imported to North America. In Russia, it occurs from the western border up to Transbaikal Region.

#### Biology

The species is common in all the parts of its range. It occurs in open biotopes in the dung of different animals, in carrion and in marmot holes.

### Aphodius (Otophorus) haemorrhoidalis

(Linnaeus,1758)

B15CF34A-A287-5EE2-90DD-D9FAAE403DB0

#### Materials

- **Type status:** Other material. **Occurrence:** recordedBy: A.S. Kurochkin; individualCount: 1; lifeStage: adult; occurrenceID: 79AC95B2-1DF7-5532-835C-A17931705FAA; **Taxon:** scientificName: Aphodiushaemorrhoidalis; kingdom: Animalia; phylum: Arthropoda; class: Insecta ; order: Coleoptera; family: Scarabaeidae; genus: Aphodius; taxonRank: species; **Location:** country: Russia; stateProvince: Orenburg; locality: Pervomaiskij Distr., 6.28 km SSW of Polyanskij vill., urochishche Pal’govo, gully bottom, forb-fescue steppe.; decimalLatitude: 51.83; decimalLongitude: 50.87; georeferenceProtocol: GPS; **Identification:** identifiedBy: L.A.Akhmetova; dateIdentified: 2023; **Event:** samplingProtocol: From feces of Marmotabobak, near burrow entrance; eventDate: 2023-05-18; **Record Level:** collectionID: urn:lsid:biocol.org:col:34969; institutionCode: ZIN; collectionCode: Coleoptera

#### Distribution

The distribution range of this species includes the whole of Europe, North Africa, the Caucasus, the Transcaucasus, Kazakhstan, Middle Asia, Afghanistan, Mongolia, north and south-western China, the Korean Peninsula and Japan; it was imported to North America. In Russia, it is distributed throughout the country.

#### Biology

The species mostly occurs in cow and horse dung, in open biotopes.

### Aphodius (Acanthobodilus) immundus

Creutzer, 1799

CD9C0BF4-A2B8-5FEE-A26E-73DAC8D5BD57

#### Materials

- **Type status:** Other material. **Occurrence:** recordedBy: A.S. Kurochkin; individualCount: 13; lifeStage: adult; occurrenceID: D2F6AB0C-F24A-5B42-8BC2-469D4D294DA2; **Taxon:** scientificName: Aphodiusimmundus; kingdom: Animalia; phylum: Arthropoda; class: Insecta ; order: Coleoptera; family: Scarabaeidae; genus: Aphodius; taxonRank: species; **Location:** country: Russia; stateProvince: Orenburg; locality: Pervomaiskij Distr., 628 km SSW of Polyanskij vill., urochishche Pal’govo, gully bottom, forb-fescue steppe.; decimalLatitude: 51.83; decimalLongitude: 50.87; georeferenceProtocol: GPS; **Identification:** identifiedBy: L.A.Akhmetova; dateIdentified: 2023; **Event:** samplingProtocol: From feces of Marmotabobak, near burrow entrance; eventDate: 2023-05-18; **Record Level:** collectionID: urn:lsid:biocol.org:col:34969; institutionCode: ZIN; collectionCode: Coleoptera
- **Type status:** Other material. **Occurrence:** recordedBy: A.S. Kurochkin; individualCount: 1; lifeStage: adult; occurrenceID: 109507E1-EDCF-55D0-A631-7758CEE97CFB; **Taxon:** scientificName: Aphodiusluridus; kingdom: Animalia; phylum: Arthropoda; class: Insecta ; order: Coleoptera; family: Scarabaeidae; genus: Aphodius; taxonRank: species; **Location:** country: Russia; stateProvince: Orenburg; locality: Pervomaiskij Distr., 7.32 km SSW of Koshkin vill., urochishche Pal’govo, gully bottom, forb-wormwood-grass steppe.; decimalLatitude: 51.82; decimalLongitude: 50.86; georeferenceProtocol: GPS; **Identification:** identifiedBy: L.A.Akhmetova; dateIdentified: 2023; **Event:** samplingProtocol: From feces of Marmotabobak, near burrow entrance; eventDate: 2023-05-19; **Record Level:** collectionID: urn:lsid:biocol.org:col:34969; institutionCode: ZIN; collectionCode: Coleoptera
- **Type status:** Other material. **Occurrence:** recordedBy: A.S. Kurochkin; individualCount: 5; lifeStage: adult; occurrenceID: 7E92C89E-A963-556C-A07A-4434163F9887; **Taxon:** scientificName: Aphodiusluridus; kingdom: Animalia; phylum: Arthropoda; class: Insecta ; order: Coleoptera; family: Scarabaeidae; genus: Aphodius; taxonRank: species; **Location:** country: Russia; stateProvince: Samara; locality: Bol’shechernigovskij distr., 3.9 km SSW of Polyanskij vill., gully bottom, forb-grass steppe.; decimalLatitude: 51.85; decimalLongitude: 50.89; georeferenceProtocol: GPS; **Identification:** identifiedBy: L.A.Akhmetova; dateIdentified: 2023; **Event:** samplingProtocol: From feces of Marmotabobak, in toilet burrow; eventDate: 2023-04-28; **Record Level:** collectionID: urn:lsid:biocol.org:col:34969; institutionCode: ZIN; collectionCode: Coleoptera
- **Type status:** Other material. **Occurrence:** recordedBy: A.S. Kurochkin; individualCount: 3; lifeStage: adult; occurrenceID: 3EA2417B-155C-52E1-A421-FE2E53B85FAC; **Taxon:** scientificName: Aphodiusluridus; kingdom: Animalia; phylum: Arthropoda; class: Insecta ; order: Coleoptera; family: Scarabaeidae; genus: Aphodius; taxonRank: species; **Location:** country: Russia; stateProvince: Samara; locality: Bol’shechernigovskij distr., 2.44 km SSW of Polyanskij vill., gully Barsuchikha, forb-fescue-feather grass steppe; decimalLatitude: 51.9; decimalLongitude: 50.93; georeferenceProtocol: GPS; **Identification:** identifiedBy: L.A.Akhmetova; dateIdentified: 2023; **Event:** samplingProtocol: From feces of Marmotabobak, in toilet burrow; eventDate: 2023-05-19; **Record Level:** collectionID: urn:lsid:biocol.org:col:34969; institutionCode: ZIN; collectionCode: Coleoptera

#### Distribution

The species occurs throughout Europe, in Morocco, Egypt, the Caucasus, the Transcaucasus, Asia Minor, Syria, Kazakhstan, middle and Central Asia. In Russia, it occurs from the western borders up to southern Yakutia.

#### Biology

The species is common in the major part of its range and prefers open biotopes. The beetles feed on horse, cow and donkey dung; they are attracted to light and occur from April to September.

### Aphodius (Acrossus) luridus

(Fabricius, 1775)

DDA56857-195D-59F7-B506-40B106059911

#### Materials

- **Type status:** Other material. **Occurrence:** recordedBy: A.S. Kurochkin; individualCount: 1; lifeStage: adult; occurrenceID: AAD30945-01B2-5763-BD6D-C70F146BA538; **Taxon:** scientificName: Aphodiusluridus; kingdom: Animalia; phylum: Arthropoda; class: Insecta ; order: Coleoptera; family: Scarabaeidae; genus: Aphodius; taxonRank: species; **Location:** country: Russia; stateProvince: Orenburg; locality: Pervomaiskij Distr., 7.32 km SSW of Koshkin vill., urochishche Pal’govo, gully bottom, forb-wormwood-grass steppe.; decimalLatitude: 51.82; decimalLongitude: 50.86; georeferenceProtocol: GPS; **Identification:** identifiedBy: L.A.Akhmetova; dateIdentified: 2023; **Event:** samplingProtocol: From feces of Marmotabobak, near burrow entrance; eventDate: 2023-05-19; **Record Level:** collectionID: urn:lsid:biocol.org:col:34969; institutionCode: ZIN; collectionCode: Coleoptera
- **Type status:** Other material. **Occurrence:** recordedBy: A.S. Kurochkin; individualCount: 5; lifeStage: adult; occurrenceID: 936AAD7B-288F-5F03-A156-933B206D5C49; **Taxon:** scientificName: Aphodiusluridus; kingdom: Animalia; phylum: Arthropoda; class: Insecta ; order: Coleoptera; family: Scarabaeidae; genus: Aphodius; taxonRank: species; **Location:** country: Russia; stateProvince: Samara; locality: Bol’shechernigovskij distr., 3.9 km SSW of Polyanskij vill., gully bottom, forb-grass steppe.; decimalLatitude: 51.85; decimalLongitude: 50.89; georeferenceProtocol: GPS; **Identification:** identifiedBy: L.A.Akhmetova; dateIdentified: 2023; **Event:** samplingProtocol: From feces of Marmotabobak, in toilet burrow; eventDate: 2023-04-28; **Record Level:** collectionID: urn:lsid:biocol.org:col:34969; institutionCode: ZIN; collectionCode: Coleoptera
- **Type status:** Other material. **Occurrence:** recordedBy: A.S. Kurochkin; individualCount: 3; lifeStage: adult; occurrenceID: 34BED712-8B19-5814-BD86-79FDC0BAD48E; **Taxon:** scientificName: Aphodiusluridus; kingdom: Animalia; phylum: Arthropoda; class: Insecta ; order: Coleoptera; family: Scarabaeidae; genus: Aphodius; taxonRank: species; **Location:** country: Russia; stateProvince: Samara; locality: Bol’shechernigovskij distr., 2.44 km SSW of Polyanskij vill., gully Barsuchikha, forb-fescue-feather grass steppe; decimalLatitude: 51.9; decimalLongitude: 50.93; georeferenceProtocol: GPS; **Identification:** identifiedBy: L.A.Akhmetova; dateIdentified: 2023; **Event:** samplingProtocol: From feces of Marmotabobak, in toilet burrow; eventDate: 2023-05-19; **Record Level:** collectionID: urn:lsid:biocol.org:col:34969; institutionCode: ZIN; collectionCode: Coleoptera

#### Distribution

The species occurs in Europe, North Africa, the Transcaucasus, Asia Minor, Kazakhstan, the mountains of Middle Asia; it was imported to North America. In Russia, it is distributed throughout the European part and occurs in south of West Siberia.

#### Biology

A coprophagous species feeding on cow, horse, donkey and sheep dung.

### Aphodius (Chilothorax) melanostictus

W. Schmidt, 1840

6911CB1D-D447-5935-9AC7-EF44D783AC76

#### Materials

- **Type status:** Other material. **Occurrence:** recordedBy: A.S. Kurochkin; individualCount: 12; lifeStage: adult; occurrenceID: EA2B55F6-EDCB-5932-B832-C06D4CCDB699; **Taxon:** scientificName: Aphodiusmelanostictus; kingdom: Animalia; phylum: Arthropoda; class: Insecta ; order: Coleoptera; family: Scarabaeidae; genus: Aphodius; taxonRank: species; **Location:** country: Russia; stateProvince: Orenburg; locality: Pervomaiskij Distr., 6.28 km SSW of Polyanskij vill., urochishche Pal’govo, gully bottom, forb-fescue steppe.; decimalLatitude: 51.83; decimalLongitude: 50.87; georeferenceProtocol: GPS; **Identification:** identifiedBy: L.A.Akhmetova; dateIdentified: 2023; **Event:** samplingProtocol: From feces of Marmotabobak, near burrow entrance; eventDate: 2023-05-18; **Record Level:** collectionID: urn:lsid:biocol.org:col:34969; institutionCode: ZIN; collectionCode: Coleoptera
- **Type status:** Other material. **Occurrence:** recordedBy: A.S. Kurochkin; individualCount: 3; lifeStage: adult; occurrenceID: B3AE70F0-579F-50EB-B984-8C8F92DDFE07; **Taxon:** scientificName: Aphodiusmelanostictus; kingdom: Animalia; phylum: Arthropoda; class: Insecta ; order: Coleoptera; family: Scarabaeidae; genus: Aphodius; taxonRank: species; **Location:** country: Russia; stateProvince: Orenburg; locality: Pervomaiskij Distr., 7.32 km SSW of Koshkin vill., urochishche Pal’govo, gully bottom, forb-wormwood-grass steppe.; decimalLatitude: 51.82; decimalLongitude: 50.86; georeferenceProtocol: GPS; **Identification:** identifiedBy: L.A.Akhmetova; dateIdentified: 2023; **Event:** samplingProtocol: From feces of Marmotabobak, near burrow entrance; eventDate: 2023-05-19; **Record Level:** collectionID: urn:lsid:biocol.org:col:34969; institutionCode: ZIN; collectionCode: Coleoptera
- **Type status:** Other material. **Occurrence:** recordedBy: A.S. Kurochkin; individualCount: 55; lifeStage: adult; occurrenceID: 322D308B-B578-55AA-8A46-5E553714E90D; **Taxon:** scientificName: Aphodiusmelanostictus; kingdom: Animalia; phylum: Arthropoda; class: Insecta ; order: Coleoptera; family: Scarabaeidae; genus: Aphodius; taxonRank: species; **Location:** country: Russia; stateProvince: Samara; locality: Bol’shechernigovskij distr., 3.9 km SSW of Polyanskij vill., gully bottom, forb-grass steppe.; decimalLatitude: 51.85; decimalLongitude: 50.89; georeferenceProtocol: GPS; **Identification:** identifiedBy: L.A.Akhmetova; dateIdentified: 2023; **Event:** samplingProtocol: From feces of Marmotabobak, in toilet burrow; eventDate: 2023-04-28; **Record Level:** collectionID: urn:lsid:biocol.org:col:34969; institutionCode: ZIN; collectionCode: Coleoptera
- **Type status:** Other material. **Occurrence:** recordedBy: A.S. Kurochkin; individualCount: 2; lifeStage: adult; occurrenceID: 9D14EABF-2629-521C-89F9-C8454C37B5DB; **Taxon:** scientificName: Aphodiusmelanostictus; kingdom: Animalia; phylum: Arthropoda; class: Insecta ; order: Coleoptera; family: Scarabaeidae; genus: Aphodius; taxonRank: species; **Location:** country: Russia; stateProvince: Samara; locality: Bol’shechernigovskij distr., 2.44 km SSW of Polyanskij vill., gully Barsuchikha, forb-fescue-feather grass steppe; decimalLatitude: 51.9; decimalLongitude: 50.93; georeferenceProtocol: GPS; **Identification:** identifiedBy: L.A.Akhmetova; dateIdentified: 2023; **Event:** samplingProtocol: From feces of Marmotabobak, in toilet burrow; eventDate: 2023-05-19; **Record Level:** collectionID: urn:lsid:biocol.org:col:34969; institutionCode: ZIN; collectionCode: Coleoptera
- **Type status:** Other material. **Occurrence:** recordedBy: A.S. Kurochkin; individualCount: 12; lifeStage: adult; occurrenceID: 02829A41-2BDE-5B6F-8D53-C4A28BAD460B; **Taxon:** scientificName: Aphodiusmelanostictus; kingdom: Animalia; phylum: Arthropoda; class: Insecta ; order: Coleoptera; family: Scarabaeidae; genus: Aphodius; taxonRank: species; **Location:** country: Russia; stateProvince: Samara; locality: Bol’shechernigovskij distr., 3.54 km SSW of Polyanskij vill., gully bottom, forb-grass steppe.; decimalLatitude: 51.86; decimalLongitude: 50.88; georeferenceProtocol: GPS; **Identification:** identifiedBy: L.A.Akhmetova; dateIdentified: 2023; **Event:** samplingProtocol: From feces of Marmotabobak, near burrow entrance; eventDate: 2023-04-28; **Record Level:** collectionID: urn:lsid:biocol.org:col:34969; institutionCode: ZIN; collectionCode: Coleoptera
- **Type status:** Other material. **Occurrence:** recordedBy: A.S. Kurochkin; individualCount: 9; lifeStage: adult; occurrenceID: F8BB424F-3BA2-58A7-822A-08AC044AA0B5; **Taxon:** scientificName: Aphodiusmelanostictus; kingdom: Animalia; phylum: Arthropoda; class: Insecta ; order: Coleoptera; family: Scarabaeidae; genus: Aphodius; taxonRank: species; **Location:** country: Russia; stateProvince: Samara; locality: Bol’shechernigovskij distr., 3.54 km SSW of Polyanskij vill., gully bottom, forb-grass steppe.; decimalLatitude: 51.86; decimalLongitude: 50.88; georeferenceProtocol: GPS; **Identification:** identifiedBy: L.A.Akhmetova; dateIdentified: 2023; **Event:** samplingProtocol: From feces of Marmotabobak, near burrow entrance; eventDate: 2023-04-28; **Record Level:** collectionID: urn:lsid:biocol.org:col:34969; institutionCode: ZIN; collectionCode: Coleoptera

#### Distribution

The species occurs in central, south and eastern Europe, North Africa, west Asia, Kazakhstan and Middle Asia. In Russia, it is distributed from the western border to Transbaikal Region, mostly in the steppe zone and the forest-steppe subzone of the deciduous forest zone.

#### Biology

The species occurs in open biotopes, in the dung of domestic animal and in marmot holes. On the territory of Russia, the beetles are active from March to October. The species is common throughout its range.

### Aphodius (Melinopterus) prodromus

(Brahm, 1790)

80101A1E-7D7A-5197-84DE-5993D7851078

#### Materials

- **Type status:** Other material. **Occurrence:** recordedBy: A.S. Kurochkin; individualCount: 1; lifeStage: adult; occurrenceID: 5744E2FA-28D7-50D4-957E-69DFEEBDA6B4; **Taxon:** scientificName: Aphodiusprodromus; kingdom: Animalia; phylum: Arthropoda; class: Insecta ; order: Coleoptera; family: Scarabaeidae; genus: Aphodius; taxonRank: species; **Location:** country: Russia; stateProvince: Orenburg; locality: Pervomaiskij Distr., 7.32 km SSW of Koshkin vill., urochishche Pal’govo, gully bottom, forb-wormwood-grass steppe.; decimalLatitude: 51.82; decimalLongitude: 50.86; georeferenceProtocol: GPS; **Identification:** identifiedBy: L.A.Akhmetova; dateIdentified: 2023; **Event:** samplingProtocol: From feces of Marmotabobak, near burrow entrance; eventDate: 2023-05-19; **Record Level:** collectionID: urn:lsid:biocol.org:col:34969; institutionCode: ZIN; collectionCode: Coleoptera

#### Distribution

The species occurs throughout Europe, in North Africa (Morocco, Algeria), the Caucasus, the Transcaucasus, Asia Minor, Syria, Lebanon, Israel, Kazakhstan, middle Asia and Mongolia; it was imported to USA and Canada. In Russia, it is distributed from the western border to Yakutia.

#### Biology

A coprophagous species feeding on dung of cows, horses and wild ungulates. In Russia, this species is common; in southern regions, it occurs from March to December.

### Aphodius (Melinopterus) punctatosulcatus

Sturm, 1805

A4955BC8-C3C2-5F2C-9F19-B3525F291235

#### Materials

- **Type status:** Other material. **Occurrence:** recordedBy: A.S. Kurochkin; individualCount: 2; lifeStage: adult; occurrenceID: F12B1842-95C6-5ADB-8E79-1816F73D9333; **Taxon:** scientificName: Aphodiuspunctatosulcatus; kingdom: Animalia; phylum: Arthropoda; class: Insecta ; order: Coleoptera; family: Scarabaeidae; genus: Aphodius; taxonRank: species; **Location:** country: Russia; stateProvince: Orenburg; locality: Pervomaiskij Distr., 6.28 km SSW of Polyanskij vill., urochishche Pal’govo, gully bottom, forb-fescue steppe.; decimalLatitude: 51.83; decimalLongitude: 50.87; georeferenceProtocol: GPS; **Identification:** identifiedBy: L.A.Akhmetova; dateIdentified: 2023; **Event:** samplingProtocol: From feces of Marmotabobak, near burrow entrance; eventDate: 2023-05-18; **Record Level:** collectionID: urn:lsid:biocol.org:col:34969; institutionCode: ZIN; collectionCode: Coleoptera

#### Distribution

The species is distributed in Europe, except for the extreme north, North Africa (Morocco, Tunisia), the Trancaucasus, west Asia, Kazakhstan and Middle Asia. In Russia, it is common in the European part, reaching the Transbaikal Region to the east.

#### Ecology

The species occurs in the dung of domestic animals and in marmot holes.

### Aphodius (Esymus) pusillus

(Herbst, 1789)

940B0760-918F-5D62-9E78-8B6DFE7AD107

#### Materials

- **Type status:** Other material. **Occurrence:** recordedBy: A.S. Kurochkin; individualCount: 2; lifeStage: adult; occurrenceID: 8AEB7875-08C8-5B80-A688-978C5781E50D; **Taxon:** scientificName: Aphodiuspusillus; kingdom: Animalia; phylum: Arthropoda; class: Insecta ; order: Coleoptera; family: Scarabaeidae; genus: Aphodius; taxonRank: species; **Location:** country: Russia; stateProvince: Orenburg; locality: Pervomaiskij Distr., 6.28 km SSW of Polyanskij vill., urochishche Pal’govo, gully bottom, forb-fescue steppe.; decimalLatitude: 51.83; decimalLongitude: 50.87; georeferenceProtocol: GPS; **Identification:** identifiedBy: L.A.Akhmetova; dateIdentified: 2023; **Event:** samplingProtocol: From feces of Marmotabobak, near burrow entrance; eventDate: 2023-05-18; **Record Level:** collectionID: urn:lsid:biocol.org:col:34969; institutionCode: ZIN; collectionCode: Coleoptera
- **Type status:** Other material. **Occurrence:** recordedBy: A.S. Kurochkin; individualCount: 2; lifeStage: adult; occurrenceID: D799945C-E37D-50EA-BEF9-93C87EDF1776; **Taxon:** scientificName: Aphodiuspusillus; kingdom: Animalia; phylum: Arthropoda; class: Insecta ; order: Coleoptera; family: Scarabaeidae; genus: Aphodius; taxonRank: species; **Location:** country: Russia; stateProvince: Samara; locality: Bol’shechernigovskij distr., 3.9 km SSW of Polyanskij vill., gully bottom, forb-grass steppe.; decimalLatitude: 51.85; decimalLongitude: 50.89; georeferenceProtocol: GPS; **Identification:** identifiedBy: L.A.Akhmetova; dateIdentified: 2023; **Event:** samplingProtocol: From feces of Marmotabobak, in toilet burrow; eventDate: 2023-04-28; **Record Level:** collectionID: urn:lsid:biocol.org:col:34969; institutionCode: ZIN; collectionCode: Coleoptera
- **Type status:** Other material. **Occurrence:** recordedBy: A.S. Kurochkin; individualCount: 2; lifeStage: adult; occurrenceID: E5DFFCBA-8F2B-5AF8-A803-A03D6F61DC70; **Taxon:** scientificName: Aphodiuspusillus; kingdom: Animalia; phylum: Arthropoda; class: Insecta ; order: Coleoptera; family: Scarabaeidae; genus: Aphodius; taxonRank: species; **Location:** country: Russia; stateProvince: Samara; locality: Bol’shechernigovskij distr., 2.44 km SSW of Polyanskij vill., gully Barsuchikha, forb-fescue-feather grass steppe; decimalLatitude: 51.9; decimalLongitude: 50.93; georeferenceProtocol: GPS; **Identification:** identifiedBy: L.A.Akhmetova; dateIdentified: 2023; **Event:** samplingProtocol: From feces of Marmotabobak, in toilet burrow; eventDate: 2023-05-19; **Record Level:** collectionID: urn:lsid:biocol.org:col:34969; institutionCode: ZIN; collectionCode: Coleoptera
- **Type status:** Other material. **Occurrence:** recordedBy: A.S. Kurochkin; individualCount: 3; lifeStage: adult; occurrenceID: 54129939-38E8-54E0-964F-63FD8F1E9D0F; **Taxon:** scientificName: Aphodiuspusillus; kingdom: Animalia; phylum: Arthropoda; class: Insecta ; order: Coleoptera; family: Scarabaeidae; genus: Aphodius; taxonRank: species; **Location:** country: Russia; stateProvince: Samara; locality: Bol’shechernigovskij distr., 3.54 km SSW of Polyanskij vill., gully bottom, forb-grass steppe.; decimalLatitude: 51.86; decimalLongitude: 50.88; georeferenceProtocol: GPS; **Identification:** identifiedBy: L.A.Akhmetova; dateIdentified: 2023; **Event:** samplingProtocol: From feces of Marmotabobak, near burrow entrance; eventDate: 2023-04-28; **Record Level:** collectionID: urn:lsid:biocol.org:col:34969; institutionCode: ZIN; collectionCode: Coleoptera

#### Distribution

The distribution range of this species includes almost the whole of Europe (except for the extreme north), the Caucasus, the Transcaucasus, Kazakhstan, Middle Asia, Asia Minor, Mongolia, north China, Korean Peninsula and Japan. In Russia, it is distributed throughout the country.

#### Biology

It is a common, locally abundant species feeding on cow, horse and sheep dung.

### Aphodius (Eudolus) quadriguttatus

(Herbst, 1783)

05F2C986-243A-542F-8E47-734DFA1AD88E

#### Materials

- **Type status:** Other material. **Occurrence:** recordedBy: A.S. Kurochkin; individualCount: 25; lifeStage: adult; occurrenceID: 6C1021DF-F310-511C-BD7C-77517E52C00A; **Taxon:** scientificName: Aphodiusquadriguttatus; kingdom: Animalia; phylum: Arthropoda; class: Insecta ; order: Coleoptera; family: Scarabaeidae; genus: Aphodius; taxonRank: species; **Location:** country: Russia; stateProvince: Orenburg; locality: Pervomaiskij Distr., 6.28 km SSW of Polyanskij vill., urochishche Pal’govo, gully bottom, forb-fescue steppe.; decimalLatitude: 51.83; decimalLongitude: 50.87; georeferenceProtocol: GPS; **Identification:** identifiedBy: L.A.Akhmetova; dateIdentified: 2023; **Event:** samplingProtocol: From feces of Marmotabobak, near burrow entrance; eventDate: 2023-05-18; **Record Level:** collectionID: urn:lsid:biocol.org:col:34969; institutionCode: ZIN; collectionCode: Coleoptera
- **Type status:** Other material. **Occurrence:** recordedBy: A.S. Kurochkin; individualCount: 6; lifeStage: adult; occurrenceID: 89BD0FA4-4EAB-54D8-AE82-5418D7630ADC; **Taxon:** scientificName: Aphodiusquadriguttatus; kingdom: Animalia; phylum: Arthropoda; class: Insecta ; order: Coleoptera; family: Scarabaeidae; genus: Aphodius; taxonRank: species; **Location:** country: Russia; stateProvince: Orenburg; locality: Pervomaiskij Distr., 7.32 km SSW of Koshkin vill., urochishche Pal’govo, gully bottom, forb-wormwood-grass steppe.; decimalLatitude: 51.82; decimalLongitude: 50.86; georeferenceProtocol: GPS; **Identification:** identifiedBy: L.A.Akhmetova; dateIdentified: 2023; **Event:** samplingProtocol: From feces of Marmotabobak, near burrow entrance; eventDate: 2023-05-19; **Record Level:** collectionID: urn:lsid:biocol.org:col:34969; institutionCode: ZIN; collectionCode: Coleoptera
- **Type status:** Other material. **Occurrence:** recordedBy: A.S. Kurochkin; individualCount: 37; lifeStage: adult; occurrenceID: 7932A530-F74A-5CF0-9C37-9C23C75EC19B; **Taxon:** scientificName: Aphodiusquadriguttatus; kingdom: Animalia; phylum: Arthropoda; class: Insecta ; order: Coleoptera; family: Scarabaeidae; genus: Aphodius; taxonRank: species; **Location:** country: Russia; stateProvince: Samara; locality: Bol’shechernigovskij distr., 3.9 km SSW of Polyanskij vill., gully bottom, forb-grass steppe.; decimalLatitude: 51.85; decimalLongitude: 50.89; georeferenceProtocol: GPS; **Identification:** identifiedBy: L.A.Akhmetova; dateIdentified: 2023; **Event:** samplingProtocol: From feces of Marmotabobak, in toilet burrow; eventDate: 2023-04-28; **Record Level:** collectionID: urn:lsid:biocol.org:col:34969; institutionCode: ZIN; collectionCode: Coleoptera
- **Type status:** Other material. **Occurrence:** recordedBy: A.S. Kurochkin; individualCount: 10; lifeStage: adult; occurrenceID: 74617261-8702-56DF-B116-A4AC9888C99D; **Taxon:** scientificName: Aphodiusquadriguttatus; kingdom: Animalia; phylum: Arthropoda; class: Insecta ; order: Coleoptera; family: Scarabaeidae; genus: Aphodius; taxonRank: species; **Location:** country: Russia; stateProvince: Samara; locality: Bol’shechernigovskij distr., 2.44 km SSW of Polyanskij vill., gully Barsuchikha, forb-fescue-feather grass steppe; decimalLatitude: 51.9; decimalLongitude: 50.93; georeferenceProtocol: GPS; **Identification:** identifiedBy: L.A.Akhmetova; dateIdentified: 2023; **Event:** samplingProtocol: From feces of Marmotabobak, in toilet burrow; eventDate: 2023-05-19; **Record Level:** collectionID: urn:lsid:biocol.org:col:34969; institutionCode: ZIN; collectionCode: Coleoptera
- **Type status:** Other material. **Occurrence:** recordedBy: A.S. Kurochkin; individualCount: 11; lifeStage: adult; occurrenceID: 5DEFA264-CB47-54B6-B012-63FC0B97DA1A; **Taxon:** scientificName: Aphodiusquadriguttatus; kingdom: Animalia; phylum: Arthropoda; class: Insecta ; order: Coleoptera; family: Scarabaeidae; genus: Aphodius; taxonRank: species; **Location:** country: Russia; stateProvince: Samara; locality: Bol’shechernigovskij distr., 3.54 km SSW of Polyanskij vill., gully bottom, forb-grass steppe.; decimalLatitude: 51.86; decimalLongitude: 50.88; georeferenceProtocol: GPS; **Identification:** identifiedBy: L.A.Akhmetova; dateIdentified: 2023; **Event:** samplingProtocol: From feces of Marmotabobak, near burrow entrance; eventDate: 2023-04-28; **Record Level:** collectionID: urn:lsid:biocol.org:col:34969; institutionCode: ZIN; collectionCode: Coleoptera
- **Type status:** Other material. **Occurrence:** recordedBy: A.S. Kurochkin; individualCount: 15; lifeStage: adult; occurrenceID: C7D9036F-0CE6-5B9C-8B85-2DC596652BF0; **Taxon:** scientificName: Aphodiusquadriguttatus; kingdom: Animalia; phylum: Arthropoda; class: Insecta ; order: Coleoptera; family: Scarabaeidae; genus: Aphodius; taxonRank: species; **Location:** country: Russia; stateProvince: Samara; locality: Bol’shechernigovskij distr., 3.54 km SSW of Polyanskij vill., gully bottom, forb-grass steppe.; decimalLatitude: 51.86; decimalLongitude: 50.88; georeferenceProtocol: GPS; **Identification:** identifiedBy: L.A.Akhmetova; dateIdentified: 2023; **Event:** samplingProtocol: From feces of Marmotabobak, near burrow entrance; eventDate: 2023-04-28; **Record Level:** collectionID: urn:lsid:biocol.org:col:34969; institutionCode: ZIN; collectionCode: Coleoptera

#### Distribution

The species is distributed in Europe, North Africa, west Asia, Kazakhstan and Middle Asia. In Russia, it mostly occurs in the Vorga Region and the Ciscaucasus.

#### Biology

A coprophagous species occurring in the dung of different animals. It prefers arid biotopes with sandy and sandy clay soils.

### Aphodius (Phalacronothus) quadrimaculatus

(Linnaeus, 1761)

4A9E5A09-3933-5D06-9A32-726636CE3108

#### Materials

- **Type status:** Other material. **Occurrence:** recordedBy: A.S. Kurochkin; individualCount: 1; lifeStage: adult; occurrenceID: 01878472-9A8A-55B9-ACB1-FDBD8C31AD6B; **Taxon:** scientificName: Aphodiusquadrimaculatus; kingdom: Animalia; phylum: Arthropoda; class: Insecta ; order: Coleoptera; family: Scarabaeidae; genus: Aphodius; taxonRank: species; **Location:** country: Russia; stateProvince: Orenburg; locality: Pervomaiskij Distr., 6.28 km SSW of Polyanskij vill., urochishche Pal’govo, gully bottom, forb-fescue steppe.; decimalLatitude: 51.83; decimalLongitude: 50.87; georeferenceProtocol: GPS; **Identification:** identifiedBy: L.A.Akhmetova; dateIdentified: 2023; **Event:** samplingProtocol: From feces of Marmotabobak, near burrow entrance; eventDate: 2023-05-18; **Record Level:** collectionID: urn:lsid:biocol.org:col:34969; institutionCode: ZIN; collectionCode: Coleoptera
- **Type status:** Other material. **Occurrence:** recordedBy: A.S. Kurochkin; individualCount: 1; lifeStage: adult; occurrenceID: 5EA2CBBB-7DAF-5695-9A5B-58E7A1939E6A; **Taxon:** scientificName: Aphodiusquadrimaculatus; kingdom: Animalia; phylum: Arthropoda; class: Insecta ; order: Coleoptera; family: Scarabaeidae; genus: Aphodius; taxonRank: species; **Location:** country: Russia; stateProvince: Samara; locality: Bol’shechernigovskij distr., 3.9 km SSW of Polyanskij vill., gully bottom, forb-grass steppe.; decimalLatitude: 51.85; decimalLongitude: 50.89; georeferenceProtocol: GPS; **Identification:** identifiedBy: L.A.Akhmetova; dateIdentified: 2023; **Event:** samplingProtocol: From feces of Marmotabobak, in toilet burrow; eventDate: 2023-04-28; **Record Level:** collectionID: urn:lsid:biocol.org:col:34969; institutionCode: ZIN; collectionCode: Coleoptera

#### Distribution

The species is widely distributed in central and south Europe; it is also known from North Africa, the Transcaucasus, Asia Minor, Syria and Turkmenistan. In Russia, it is known from Samara and Astrakhan Provinces.

#### Biology

The species occurs in cow dung.

### Aphodius (Biralus) satellitus

(Herbst, 1789)

A048635D-9C05-59A9-89A9-536FCFC83034

#### Materials

- **Type status:** Other material. **Occurrence:** recordedBy: A.S. Kurochkin; individualCount: 1; lifeStage: adult; occurrenceID: 7CCA8317-A974-5765-9D12-40F3E38C60D7; **Taxon:** scientificName: Aphodiussatellitus; kingdom: Animalia; phylum: Arthropoda; class: Insecta ; order: Coleoptera; family: Scarabaeidae; genus: Aphodius; taxonRank: species; **Location:** country: Russia; stateProvince: Orenburg; locality: Pervomaiskij Distr., 6.28 km SSW of Polyanskij vill., urochishche Pal’govo, gully bottom, forb-fescue steppe.; decimalLatitude: 51.83; decimalLongitude: 50.87; georeferenceProtocol: GPS; **Identification:** identifiedBy: L.A.Akhmetova; dateIdentified: 2023; **Event:** samplingProtocol: From feces of Marmotabobak, near burrow entrance; eventDate: 2023-05-18; **Record Level:** collectionID: urn:lsid:biocol.org:col:34969; institutionCode: ZIN; collectionCode: Coleoptera
- **Type status:** Other material. **Occurrence:** recordedBy: A.S. Kurochkin; individualCount: 2; lifeStage: adult; occurrenceID: 53CC3420-E2CF-5B46-81A1-15EED3811248; **Taxon:** scientificName: Aphodiussatellitus; kingdom: Animalia; phylum: Arthropoda; class: Insecta ; order: Coleoptera; family: Scarabaeidae; genus: Aphodius; taxonRank: species; **Location:** country: Russia; stateProvince: Samara; locality: Bol’shechernigovskij distr., 3.9 km SSW of Polyanskij vill., gully bottom, forb-grass steppe.; decimalLatitude: 51.85; decimalLongitude: 50.89; georeferenceProtocol: GPS; **Identification:** identifiedBy: L.A.Akhmetova; dateIdentified: 2023; **Event:** samplingProtocol: From feces of Marmotabobak, in toilet burrow; eventDate: 2023-04-28; **Record Level:** collectionID: urn:lsid:biocol.org:col:34969; institutionCode: ZIN; collectionCode: Coleoptera
- **Type status:** Other material. **Occurrence:** recordedBy: A.S. Kurochkin; individualCount: 1; lifeStage: adult; occurrenceID: D07EE430-D3D0-5A27-9CF8-C993F64F2C43; **Taxon:** scientificName: Aphodiussatellitus; kingdom: Animalia; phylum: Arthropoda; class: Insecta ; order: Coleoptera; family: Scarabaeidae; genus: Aphodius; taxonRank: species; **Location:** country: Russia; stateProvince: Samara; locality: Bol’shechernigovskij distr., 2.44 km SSW of Polyanskij vill., gully Barsuchikha, forb-fescue-feather grass steppe; decimalLatitude: 51.9; decimalLongitude: 50.93; georeferenceProtocol: GPS; **Identification:** identifiedBy: L.A.Akhmetova; dateIdentified: 2023; **Event:** samplingProtocol: From feces of Marmotabobak, in toilet burrow; eventDate: 2023-05-19; **Record Level:** collectionID: urn:lsid:biocol.org:col:34969; institutionCode: ZIN; collectionCode: Coleoptera

#### Distribution

The species occurs in central andeEastern Europe, the Transcaucasus, Middle Asia. In Russia, it is mainly distributed in northern Caucasus and the Volga Region.

#### Biology

The species occurs in cow and horse dung, mostly in open biotopes.

### Aphodius (Trichonotulus) scrofa

(Fabricius, 1787)

F1C43328-B44A-5E79-A6BD-F696A5B9033B

#### Materials

- **Type status:** Other material. **Occurrence:** recordedBy: A.S. Kurochkin; individualCount: 6; lifeStage: adult; occurrenceID: 7B8E6E5A-053F-5359-B102-1F9D8DE968CA; **Taxon:** scientificName: Aphodiusscrofa; kingdom: Animalia; phylum: Arthropoda; class: Insecta ; order: Coleoptera; family: Scarabaeidae; genus: Aphodius; taxonRank: species; **Location:** country: Russia; stateProvince: Orenburg; locality: Pervomaiskij Distr., 6.28 km SSW of Polyanskij vill., urochishche Pal’govo, gully bottom, forb-fescue steppe.; decimalLatitude: 51.83; decimalLongitude: 50.87; georeferenceProtocol: GPS; **Identification:** identifiedBy: L.A.Akhmetova; dateIdentified: 2023; **Event:** samplingProtocol: From feces of Marmotabobak, near burrow entrance; eventDate: 2023-05-18; **Record Level:** collectionID: urn:lsid:biocol.org:col:34969; institutionCode: ZIN; collectionCode: Coleoptera
- **Type status:** Other material. **Occurrence:** recordedBy: A.S. Kurochkin; individualCount: 4; lifeStage: adult; occurrenceID: D67C18F8-46D7-5028-8D46-49E107055B5E; **Taxon:** scientificName: Aphodiusscrofa; kingdom: Animalia; phylum: Arthropoda; class: Insecta ; order: Coleoptera; family: Scarabaeidae; genus: Aphodius; taxonRank: species; **Location:** country: Russia; stateProvince: Samara; locality: Bol’shechernigovskij distr., 3.9 km SSW of Polyanskij vill., gully bottom, forb-grass steppe.; decimalLatitude: 51.85; decimalLongitude: 50.89; georeferenceProtocol: GPS; **Identification:** identifiedBy: L.A.Akhmetova; dateIdentified: 2023; **Event:** samplingProtocol: From feces of Marmotabobak, in toilet burrow; eventDate: 2023-04-28; **Record Level:** collectionID: urn:lsid:biocol.org:col:34969; institutionCode: ZIN; collectionCode: Coleoptera

#### Distribution

The species occurs in Europe (up to Finland in the north), North Africa (Morocco), the Caucasus, the Transcaucasus, Asia Minor, Kazakhstan, Middle Asia, Afghanistan, Mongolia, China, North Korea. It was imported to Canada and the USA. In Russia, the species is mostly distributed in the forest-steppe subzone of the deciduous forest zone from the western border to the Amur Region.

#### Biology

A coprophagous species occurring in ungulate dung.

### Aphodius (Nialus) varians

Duftschmid, 1805

EF2BA2AD-7725-54D3-BE8D-77DE9509EC0D

#### Materials

- **Type status:** Other material. **Occurrence:** recordedBy: A.S. Kurochkin; individualCount: 13; lifeStage: adult; occurrenceID: 70EAF21A-425D-56B1-B2EE-E6C05052EFAF; **Taxon:** scientificName: Aphodiusvarians; kingdom: Animalia; phylum: Arthropoda; class: Insecta ; order: Coleoptera; family: Scarabaeidae; genus: Aphodius; taxonRank: species; **Location:** country: Russia; stateProvince: Orenburg; locality: Pervomaiskij Distr., 6.28 km SSW of Polyanskij vill., urochishche Pal’govo, gully bottom, forb-fescue steppe.; decimalLatitude: 51.83; decimalLongitude: 50.87; georeferenceProtocol: GPS; **Identification:** identifiedBy: L.A.Akhmetova; dateIdentified: 2023; **Event:** samplingProtocol: From feces of Marmotabobak, near burrow entrance; eventDate: 2023-05-18; **Record Level:** collectionID: urn:lsid:biocol.org:col:34969; institutionCode: ZIN; collectionCode: Coleoptera

#### Distribution

The distribution range of this species includes central and southern Europe, North Africa, the Transcaucasus, Asia Minor, Kazakhstan and Middle Asia. In Russia, it is mostly distributed in the steppe zone and forest-steppe subzone of the zone of deciduous forest from the western border to South Siberia.

#### Biology

The species occurs in diverse biotopes, in cow and horse dung and in riverside debris.

### Onthophagus (Palaeonthophagus) semicornis

(Panzer, 1798)

7A86D34E-E6EA-5ACC-BB73-FEE43F05F7AE

#### Distribution

The species is distributed in southern part of western and central Palaearctic.

#### Ecology

The species mostly occurs in steppe biotopes. A generalist coprophage, feeding on ungulate dung, faeces of rodents and birds.

### Onthophagus (Palaeonthophagus) nuchicornis

(Linnaeus, 1758)

AC10555E-BEEC-50EC-9BBB-01D2E753AE51

#### Materials

- **Type status:** Other material. **Occurrence:** recordedBy: A.S. Kurochkin; individualCount: 1; lifeStage: adult; occurrenceID: 52A7F45A-AD03-557F-90D1-B007889C9F64; **Taxon:** scientificName: Onthophagusnuchicornis; kingdom: Animalia; phylum: Arthropoda; class: Insecta ; order: Coleoptera; family: Scarabaeidae; genus: Onthophagus; taxonRank: species; **Location:** country: Russia; stateProvince: Samara; locality: Bol’shechernigovskij distr., 2.44 km SSW of Polyanskij vill., gully Barsuchikha, forb-fescue-feather grass steppe; decimalLatitude: 51.9; decimalLongitude: 50.93; georeferenceProtocol: GPS; **Identification:** identifiedBy: A.V.Frolov; dateIdentified: 2023; **Event:** samplingProtocol: From feces of Marmotabobak, in toilet burrow; eventDate: 2023-05-19; **Record Level:** collectionID: urn:lsid:biocol.org:col:34969; institutionCode: ZIN; collectionCode: Coleoptera
- **Type status:** Other material. **Occurrence:** recordedBy: A.S. Kurochkin; individualCount: 1; lifeStage: adult; occurrenceID: 9CE44B0E-8341-5545-B1D3-ECA1CDE97478; **Taxon:** scientificName: Onthophagusnuchicornis; kingdom: Animalia; phylum: Arthropoda; class: Insecta ; order: Coleoptera; family: Scarabaeidae; genus: Onthophagus; taxonRank: species; **Location:** country: Russia; stateProvince: Samara; locality: Bol’shechernigovskij distr., 3.54 km SSW of Polyanskij vill., gully bottom, forb-grass steppe.; decimalLatitude: 51.86; decimalLongitude: 50.88; georeferenceProtocol: GPS; **Identification:** identifiedBy: A.V.Frolov; dateIdentified: 2023; **Event:** samplingProtocol: From feces of Marmotabobak, near burrow entrance; eventDate: 2023-04-28; **Record Level:** collectionID: urn:lsid:biocol.org:col:34969; institutionCode: ZIN; collectionCode: Coleoptera
- **Type status:** Other material. **Occurrence:** recordedBy: A.S. Kurochkin; individualCount: 2; lifeStage: adult; occurrenceID: 4E857A31-DEBA-5C11-AFA8-DE2C847F3E95; **Taxon:** scientificName: Onthophagusnuchicornis; kingdom: Animalia; phylum: Arthropoda; class: Insecta ; order: Coleoptera; family: Scarabaeidae; genus: Onthophagus; taxonRank: species; **Location:** country: Russia; stateProvince: Samara; locality: Bol’shechernigovskij distr., 3.9 km SSW of Polyanskij vill., gully bottom, forb-grass steppe.; decimalLatitude: 51.85; decimalLongitude: 50.89; georeferenceProtocol: GPS; **Identification:** identifiedBy: A.V.Frolov; dateIdentified: 2023; **Event:** samplingProtocol: From feces of Marmotabobak, in toilet burrow; eventDate: 2023-04-28; **Record Level:** collectionID: urn:lsid:biocol.org:col:34969; institutionCode: ZIN; collectionCode: Coleoptera
- **Type status:** Other material. **Occurrence:** recordedBy: A.S. Kurochkin; individualCount: 5; lifeStage: adult; occurrenceID: 756B483A-BE38-526B-8F76-313363050D32; **Taxon:** scientificName: Onthophagusnuchicornis; kingdom: Animalia; phylum: Arthropoda; class: Insecta ; order: Coleoptera; family: Scarabaeidae; genus: Onthophagus; taxonRank: species; **Location:** country: Russia; stateProvince: Orenburg; locality: Pervomaiskij Distr., 7.32 km SSW of Koshkin vill., urochishche Pal’govo, gully bottom, forb-wormwood-grass steppe.; decimalLatitude: 51.82; decimalLongitude: 50.86; georeferenceProtocol: GPS; **Identification:** identifiedBy: A.V.Frolov; dateIdentified: 2023; **Event:** samplingProtocol: From feces of Marmotabobak, near burrow entrance; eventDate: 2023-05-19; **Record Level:** collectionID: urn:lsid:biocol.org:col:34969; institutionCode: ZIN; collectionCode: Coleoptera

#### Distribution

The spcies is widely distributed in the Palaearctic Region, introduced in North America. In Russia, it occurs throughout the European part, in West and Central Siberia.

#### Biology

A generalist coprophagous species feeding mostly on cattle dung. It was also registered feeding on carrion and rotten food.

### Onthophagus (Palaeonthophagus) vacca

(Linnaeus, 1767)

2C3B96AB-ACF4-5A69-85A5-57AFD2F4EE0C

#### Materials

- **Type status:** Other material. **Occurrence:** recordedBy: A.S. Kurochkin; individualCount: 1; lifeStage: adult; occurrenceID: A09B54B0-8617-5A60-938B-BD207CB3C8DB; **Taxon:** scientificName: Onthophagusvacca; kingdom: Animalia; phylum: Arthropoda; class: Insecta ; order: Coleoptera; family: Scarabaeidae; genus: Onthophagus; taxonRank: species; **Location:** country: Russia; stateProvince: Samara; locality: Bol’shechernigovskij distr., 2.44 km SSW of Polyanskij vill., gully Barsuchikha, forb-fescue-feather grass steppe; decimalLatitude: 51.9; decimalLongitude: 50.93; georeferenceProtocol: GPS; **Identification:** identifiedBy: A.V.Frolov; dateIdentified: 2023; **Event:** samplingProtocol: From feces of Marmotabobak, in toilet burrow; eventDate: 2023-05-19; **Record Level:** collectionID: urn:lsid:biocol.org:col:34969; institutionCode: ZIN; collectionCode: Coleoptera
- **Type status:** Other material. **Occurrence:** recordedBy: A.S. Kurochkin; individualCount: 1; lifeStage: adult; occurrenceID: 4FE4DBE5-6A72-58D9-AFDE-26FAE8701DE7; **Taxon:** scientificName: Onthophagusvacca; kingdom: Animalia; phylum: Arthropoda; class: Insecta ; order: Coleoptera; family: Scarabaeidae; genus: Onthophagus; taxonRank: species; **Location:** country: Russia; stateProvince: Samara; locality: Bol’shechernigovskij distr., 3.54 km SSW of Polyanskij vill., gully bottom, forb-grass steppe.; decimalLatitude: 51.86; decimalLongitude: 50.88; georeferenceProtocol: GPS; **Identification:** identifiedBy: A.V.Frolov; dateIdentified: 2023; **Event:** samplingProtocol: From feces of Marmotabobak, near burrow entrance; eventDate: 2023-04-28; **Record Level:** collectionID: urn:lsid:biocol.org:col:34969; institutionCode: ZIN; collectionCode: Coleoptera
- **Type status:** Other material. **Occurrence:** recordedBy: A.S. Kurochkin; individualCount: 3; lifeStage: adult; occurrenceID: B5B75DF7-224B-5EA7-99E8-198D750574D5; **Taxon:** scientificName: Onthophagusvacca; kingdom: Animalia; phylum: Arthropoda; class: Insecta ; order: Coleoptera; family: Scarabaeidae; genus: Onthophagus; taxonRank: species; **Location:** country: Russia; stateProvince: Orenburg; locality: Pervomaiskij Distr., 6.28 km SSW of Polyanskij vill., urochishche Pal’govo, gully bottom, forb-fescue steppe.; decimalLatitude: 51.83; decimalLongitude: 50.87; georeferenceProtocol: GPS; **Identification:** identifiedBy: A.V.Frolov; dateIdentified: 2023; **Event:** samplingProtocol: From feces of Marmotabobak, near burrow entrance; eventDate: 2023-05-18; **Record Level:** collectionID: urn:lsid:biocol.org:col:34969; institutionCode: ZIN; collectionCode: Coleoptera
- **Type status:** Other material. **Occurrence:** recordedBy: A.S. Kurochkin; individualCount: 6; lifeStage: adult; occurrenceID: 0F9494E5-8F29-5E76-A3D7-294C8D5846A7; **Taxon:** scientificName: Onthophagusvacca; kingdom: Animalia; phylum: Arthropoda; class: Insecta ; order: Coleoptera; family: Scarabaeidae; genus: Onthophagus; taxonRank: species; **Location:** country: Russia; stateProvince: Orenburg; locality: Pervomaiskij Distr., 7.32 km SSW of Koshkin vill., urochishche Pal’govo, gully bottom, forb-wormwood-grass steppe.; decimalLatitude: 51.82; decimalLongitude: 50.86; georeferenceProtocol: GPS; **Identification:** identifiedBy: A.V.Frolov; dateIdentified: 2023; **Event:** samplingProtocol: From feces of Marmotabobak, near burrow entrance; eventDate: 2023-05-19; **Record Level:** collectionID: urn:lsid:biocol.org:col:34969; institutionCode: ZIN; collectionCode: Coleoptera

#### Distribution

A western Palaearctic species. In Russia, it is common in the central and southern European part, reaching southern Urals Mountains in the east.

#### Biology

A generalist coprophagous species feeding mostly on cattle dung.

### Onthophagus (Palaeonthophagus) leucostigma

Steven, 1811

5B822992-38F9-50B7-A745-1941160320AF

#### Materials

- **Type status:** Other material. **Occurrence:** recordedBy: A.S. Kurochkin; individualCount: 9; lifeStage: adult; occurrenceID: CBAEC39F-94A2-5569-9D93-8812145FCB69; **Taxon:** scientificName: Onthophagusleucostigma; kingdom: Animalia; phylum: Arthropoda; class: Insecta ; order: Coleoptera; family: Scarabaeidae; genus: Onthophagus; taxonRank: species; **Location:** country: Russia; stateProvince: Orenburg; locality: Pervomaiskij Distr., 6.28 km SSW of Polyanskij vill., urochishche Pal’govo, gully bottom, forb-fescue steppe.; decimalLatitude: 51.83; decimalLongitude: 50.87; georeferenceProtocol: GPS; **Identification:** identifiedBy: A.V.Frolov; dateIdentified: 2023; **Event:** samplingProtocol: From feces of Marmotabobak, near burrow entrance; eventDate: 2023-05-18; **Record Level:** collectionID: urn:lsid:biocol.org:col:34969; institutionCode: ZIN; collectionCode: Coleoptera

#### Distribution

Southern Ukraine, southern European Russia up to Volga Basin in the east, north-western Kazakhstan.

#### Biology

A nidicolous species inhabiting burrows of steppe rodents. It was also registered feeding on sheep and cattle dung.

### Caccobius schreberi

(Linnaeus, 1767)

802FB2CD-C491-5E6B-B938-88AF200BB88E

#### Materials

- **Type status:** Other material. **Occurrence:** recordedBy: A.S. Kurochkin; individualCount: 2; lifeStage: adult; occurrenceID: D8047A27-CD3B-5A1C-8486-E4FB605EEEC2; **Taxon:** scientificName: Caccobiusschreberi; kingdom: Animalia; phylum: Arthropoda; class: Insecta ; order: Coleoptera; family: Scarabaeidae; genus: Caccobius; taxonRank: species; **Location:** country: Russia; stateProvince: Samara; locality: Bol’shechernigovskij distr., 3.9 km SSW of Polyanskij vill., gully bottom, forb-grass steppe.; decimalLatitude: 51.85; decimalLongitude: 50.89; georeferenceProtocol: GPS; **Identification:** identifiedBy: A.V.Frolov; dateIdentified: 2023; **Event:** samplingProtocol: From feces of Marmotabobak, in toilet burrow; eventDate: 2023-04-28; **Record Level:** collectionID: urn:lsid:biocol.org:col:34969; institutionCode: ZIN; collectionCode: Coleoptera

#### Distribution

The species is distributed in southern and middle Europe, northern Africa, Asia Minor and Iranian Plateau.

#### Biology

A common, sometimes abundant generalist coprophage feeding mostly on cattle, horse and sheep dung.

### Sisyphus schaefferi

(Linnaeus, 1758)

E48BE5E4-C3EB-5140-B169-B9BA09F61EF1

#### Materials

- **Type status:** Other material. **Occurrence:** recordedBy: A.S. Kurochkin; individualCount: 1; lifeStage: adult; occurrenceID: 99EC5DC7-7880-5804-BE51-C76736CA8597; **Taxon:** scientificName: Sisyphusschaefferi; kingdom: Animalia; phylum: Arthropoda; class: Insecta ; order: Coleoptera; family: Scarabaeidae; genus: Sisyphus; taxonRank: species; **Location:** country: Russia; stateProvince: Samara; locality: Bol’shechernigovskij distr., 2.44 km SSW of Polyanskij vill., gully Barsuchikha, forb-fescue-feather grass steppe; decimalLatitude: 51.9; decimalLongitude: 50.93; georeferenceProtocol: GPS; **Identification:** identifiedBy: A.V. Frolov; dateIdentified: 2023; **Event:** samplingProtocol: From feces of Marmotabobak, in toilet burrow; eventDate: 2023-05-19; **Record Level:** collectionID: urn:lsid:biocol.org:col:34969; institutionCode: ZIN; collectionCode: Coleoptera
- **Type status:** Other material. **Occurrence:** recordedBy: A.S. Kurochkin; individualCount: 5; lifeStage: adult; occurrenceID: 47D23F9B-CBB8-5FAA-91DC-23277890E809; **Taxon:** scientificName: Sisyphusschaefferi; kingdom: Animalia; phylum: Arthropoda; class: Insecta ; order: Coleoptera; family: Scarabaeidae; genus: Sisyphus; taxonRank: species; **Location:** country: Russia; stateProvince: Samara; locality: Bol’shechernigovskij distr., 3.53 km SSW of Polyanskij vill., gully bottom, forb-grass steppe.; decimalLatitude: 51.86; decimalLongitude: 50.88; georeferenceProtocol: GPS; **Identification:** identifiedBy: A.V.Frolov; dateIdentified: 2023; **Event:** samplingProtocol: From feces of Marmotabobak, near burrow entrance; eventDate: 2023-05-19; **Record Level:** collectionID: urn:lsid:biocol.org:col:34969; institutionCode: ZIN; collectionCode: Coleoptera
- **Type status:** Other material. **Occurrence:** recordedBy: A.S. Kurochkin; individualCount: 3; lifeStage: adult; occurrenceID: 5C76CCBD-6163-5CB9-82DC-2DC380FFC75F; **Taxon:** scientificName: Sisyphusschaefferi; kingdom: Animalia; phylum: Arthropoda; class: Insecta ; order: Coleoptera; family: Scarabaeidae; genus: Sisyphus; taxonRank: species; **Location:** country: Russia; stateProvince: Samara; locality: Bol’shechernigovskij distr., 3.9 km SSW of Polyanskij vill., gully bottom, forb-grass steppe.; decimalLatitude: 51.85; decimalLongitude: 50.89; georeferenceProtocol: GPS; **Identification:** identifiedBy: A.V.Frolov; dateIdentified: 2023; **Event:** samplingProtocol: From feces of Marmotabobak, in toilet burrow; eventDate: 2023-04-28; **Record Level:** collectionID: urn:lsid:biocol.org:col:34969; institutionCode: ZIN; collectionCode: Coleoptera
- **Type status:** Other material. **Occurrence:** recordedBy: A.S. Kurochkin; individualCount: 8; lifeStage: adult; occurrenceID: 36363A19-4A50-5B05-BCCB-760783CCB9A4; **Taxon:** scientificName: Sisyphusschaefferi; kingdom: Animalia; phylum: Arthropoda; class: Insecta ; order: Coleoptera; family: Scarabaeidae; genus: Sisyphus; taxonRank: species; **Location:** country: Russia; stateProvince: Orenburg; locality: Pervomaiskij Distr., 6.28 km SSW of Polyanskij vill., urochishche Pal’govo, gully bottom, forb-fescue steppe.; decimalLatitude: 51.83; decimalLongitude: 50.87; georeferenceProtocol: GPS; **Identification:** identifiedBy: A.V.Frolov; dateIdentified: 2023; **Event:** samplingProtocol: From feces of Marmotabobak, near burrow entrance; eventDate: 2023-05-18; **Record Level:** collectionID: urn:lsid:biocol.org:col:34969; institutionCode: ZIN; collectionCode: Coleoptera

#### Distribution

The species is widely distributed in the southern Palaearctic.

#### Biology

The species mostly occurs in steppe and semi-desert biotopes. A generalist coprophage, feeding mostly on ungulate dung.

### Euoniticellus fulvus

Goeze, 1777

DB0444EB-F02D-58F7-A02D-54AC9EAA51F2

#### Materials

- **Type status:** Other material. **Occurrence:** recordedBy: A.S. Kurochkin; individualCount: 1; lifeStage: adult; occurrenceID: DB3BE184-211E-5CA7-AA85-13909FC44C7B; **Taxon:** scientificName: Euoniticellusfulvus; kingdom: Animalia; phylum: Arthropoda; class: Insecta ; order: Coleoptera; family: Scarabaeidae; genus: Euoniticellus; taxonRank: species; **Location:** country: Russia; stateProvince: Orenburg; locality: Pervomaiskij Distr., 6.28 km SSW of Polyanskij vill., urochishche Pal’govo, gully bottom, forb-fescue steppe.; decimalLatitude: 51.83; decimalLongitude: 50.87; georeferenceProtocol: GPS; **Identification:** identifiedBy: A.V.Frolov; dateIdentified: 2023; **Event:** samplingProtocol: From feces of Marmotabobak, near burrow entrance; eventDate: 2023-05-18; **Record Level:** collectionID: urn:lsid:biocol.org:col:34969; institutionCode: ZIN; collectionCode: Coleoptera

#### Distribution

The species is distributed in southern Europe, Asia Minor and western Central Asia.

#### Biology

A generalist coprophagous species feeding mostly on cattle and horse dung.

## Analysis

The collected specimens belonged to 28 species of two coprophagous subfamilies – Aphodiinae and Scarabaeinae (Table 2). The former subfamily was represented by the genus *Aphodius* and comprised majority of species. Scarabaeinae comprised seven species of four genera. *Onthophagus* was represented by four species, while *Caccobius*, *Euoniticellus* and *Sisyphus* – by one species each. The number of species per locality varied significantly, from 22 species in locality 6 to only three in locality 2. Of the 28 species encountered, only two *Aphodius* species belonged to specialist nidicolous, associated with ground squirrels, while the great majority belong to generalist coprophages which apparently do not utilise marmot faeces as a primary food source (but see below regarding *O.leucostigma*). Seven species were not previously recorded as consumers of marmot faeces.

## Discussion

In the open terrestrial biomes of the Holarctic realm, ground squirrels of the tribe Marmotini are commonly recognised as keystone species, having a major impact on their ecological communities. Burrow-dwelling rodents are considered ecosystem engineer species in steppes where they shape the plant species composition and diversity (Valkó et al. 2021). It was demonstrated that the European ground squirrel *Spermophiluscitellus* (Linnaeus, 1766) helps to maintain heterogeneity in temperate grassland ecosystems (Lindtner et al. 2020, Lindtner et al. 2018). Ground squirrels are a focal group for conservation efforts in many countries (Caro 2010).

There exists a nidicolous fauna of insects that are associated with burrowing rodents and live in their burrows. One of the prominent components of this fauna are the Scarabaeidae dung beetles that feed on rodent faeces and those larvae develop inside the burrows. Zunino and Halffter (2007) reviewed the information of associations of *Onthophagus* Latreille, 1802, species with vertebrate burrows and reported a reasonable number of species associated with marmots and ecologically similar rodents. Similar synoptic studies for Aphodiinae or *Aphodius* Hellwig, 1798, are not available, but considering that Aphodiinae, especially the mega-diverse genus *Aphodius*, dominate dung-beetle communities in the many regions where ground squirrels are distributed, it is expected a similarly diverse nidicolous *Aphodius* fauna.

In studying of the association between burrowing rodents and scarab beetles, one should distinguish between trophic association and development association. The former implies that a certain beetle species feeds on excrements of a certain rodent species. It may be a specialist coprophage, feeding only on excrements of one species or that prefers faeces of a certain rodent species, but may occasionally feed on faeces of other animals and last, but not least a generalist coprophage that feeds on faeces of different animals occurring within its range. Development association refers to specialist nidicolous species that depend, during at least one of its development stage, on the presence of the rodent species and cannot develop without its burrows. In this respect, feeding of a beetles species on the rodent faeces does not necessarily mean that it is a nidicolus species and, vice versa, a species found inside a burrow did not necessarily came to the burrow attracted to rodent faeces.

The present study is focused on the trophic association of scarab beetles and steppe marmots. During the survey, we encountered a reasonably large number of dung beetle species attracted to steppe marmot faeces. Most of these species, 26 of 28, belong to generalist coprophages (Table 2).

Several researchers studied beetles, including Scarabaeidae, associated with *Marmota* species and collected from inside burrows or immediately near burrow openings. For example, two specialist nidicolous species of *Aphodius*, *A.isajevi* Kabakov, 1994 and *A.exilimanus* Kabakov, 1994, were described from steppe marmot burrows in similar steppe biotopes in southern Ul’yanovsk Province, Russia, some 230 km NW of the survey area (Kabakov 1994). These beetles were collected in the same period as our survey (second half of May) and Isajev (1995) noted that both species were rather common and locally abundant, but only occurred in native colonies of steppe marmots; they were not found in the colonies of re-introduced marmot.

Similar results were obtained by Egorov (1997) who studied insects associated with two steppe marmot colonies in Chuvashiya (some 500 km NW of the survey area). In the native, relict colony, he found 10 Aphodiinae and two Scarabaeinae species, while, in the re-acclimatised colony, six Aphodiinae and one Scarabaeinae species. In the first colony, specialist nidicolous species, namely *A.arenarius* Olivier, 1789 (= *A.putridus* (Fourcroy, 1785)), *A.rotundangulus* Reitter, 1900 and *O.vitulus* (Fabricius, 1776), dominated the Scarabaeidae community. While in the re-acclimatised colony, all species belonged to generalist coprophages.

The exact status of the examined colonies of steppe marmot is not clear. Bibikov et al. (1990) wrote that before the increased anthropogenic pressure, steppe marmots were abundant in steppe biotopes in Russia but, due to almost continuous ploughing of native steppes and unrestricted hunting, the species was almost exterminated in Russia by the beginning of the 20^th^ century. In 1970s-1990s, steppe marmots were re-introduced in large territories of its former range, including the southern part of Samara Province (Bibikov et al. 1990). Naumov et al. (2013) wrote that, by the 1950s in Samara Province, native colonies of the steppe marmot existed in a few distant areas, including Bol’shechernigovskij District, but noted that the detailed information about the distribution of the species at that time is lacking. After a comprehensive survey of the steppe marmot in Samara Province, they found a metapopulation with 22 colonies and 118 families in Bol’sheglushitskij and Bol’shechernigovskij Districts. They also concluded that the majority of the colonies were at the earlier stages of the introduction (Naumov et al. 2013).

Our results are congruent with the above-mentioned; however they are somewhat intermediate between the results that previous researchers reported for native and re-acclimatised colonies of steppe marmots. The fauna of the associated dung-beetles in the surveyed area did not include only generalist coprophages, as was previously reported for all studied re-acclimatised colonies (Isajev 1995, Egorov 1997). We found two specialist nidicolous species of *Aphodius*, *A.arenarius* and *A.citellorum*, in locality 4, but they were represented by only one and six specimens, respectively. We also found *O.leucostigma* in locality 5. This species is not apparently strictly nidicolous, but is known to prefer living in burrows of ground squirrels (Carpaneto and Pittino 1998, Kabakov 2006). Therefore, we did not encounter the fauna characteristic for native marmot colonies in terms of both abundance and species richness. Available data (e.g. Frolov and Akhmetova (2013), Isajev (1995)) suggest that nidicolous species of *Aphodius* are rather common or abundant in suitable habitats. Provided that the marmot colonies are native, in the studied area, one would expect at least a few other nidicolous species, namely *A.rotundangulus*, *A.isajevi*, *A.exilimanus* and *O.vitulus*.

It should be noted that the surveyed area lies within distribution ranges of two souslik species, *Spermophiluspygmaeus* (Pallas) and *S.major* (Pallas) (Dudnikov et al. 2022), which potentially can support nidicolous fauna. They were, however, not encountered during our study.

Our results suggest that the steppe marmot colonies in the surveyed area are a result of a recent (apparently a few decades old) dispersion from southern regions with stable populations. Further monitoring of the nidicolous species will probably show re-establishing of the fauna associated with marmots and support the assumption of the important role of ground squirrels in biodiversity of the steppe biotopes as well as agricultural lands.

In the present contribution, we also report our findings about trophic associations of beetles and marmots as a set of nanopublications. Nanopublications are small data containers represented by named RDF graphs that can be automatically interpreted and aggregated (Kuhn et al. 2016). One of the possible uses of nanopublications in biodiversity is communicating new information in a standardised way (Dimitrova et al. 2021). We think that such nanopublication may be especially useful in accumulating and analysing data in food webs research.

## Supplementary Material

XML Treatment for Aphodius (Plagiogonus) arenarius

XML Treatment for Aphodius (Phalacronothus) biguttatus

XML Treatment for Aphodius (Acrossus) bimaculatus

XML Treatment for Aphodius (Phalacronothus) citellorum

XML Treatment for Aphodius (Euorodalus) coenosus

XML Treatment for Aphodius (Chilothorax) distinctus

XML Treatment for Aphodius (Colobopterus) erraticus

XML Treatment for Aphodius (Aphodius) fimetarius

XML Treatment for Aphodius (Calamosternus) granarius

XML Treatment for Aphodius (Otophorus) haemorrhoidalis

XML Treatment for Aphodius (Acanthobodilus) immundus

XML Treatment for Aphodius (Acrossus) luridus

XML Treatment for Aphodius (Chilothorax) melanostictus

XML Treatment for Aphodius (Melinopterus) prodromus

XML Treatment for Aphodius (Melinopterus) punctatosulcatus

XML Treatment for Aphodius (Esymus) pusillus

XML Treatment for Aphodius (Eudolus) quadriguttatus

XML Treatment for Aphodius (Phalacronothus) quadrimaculatus

XML Treatment for Aphodius (Biralus) satellitus

XML Treatment for Aphodius (Trichonotulus) scrofa

XML Treatment for Aphodius (Nialus) varians

XML Treatment for Onthophagus (Palaeonthophagus) semicornis

XML Treatment for Onthophagus (Palaeonthophagus) nuchicornis

XML Treatment for Onthophagus (Palaeonthophagus) vacca

XML Treatment for Onthophagus (Palaeonthophagus) leucostigma

XML Treatment for Caccobius schreberi

XML Treatment for Sisyphus schaefferi

XML Treatment for Euoniticellus fulvus

## Figures and Tables

**Table:** 

Nanopublication	Creator	Date

**Table 1.** Characterisation of the collecting localities.

**No**	**Photo**	**Locality**	**Date**	**Coordinates**	**Characterization**
1	Fig. 3	Bol’shechernigovskij Distr., 2.44 km SSW of Polyanskij Vill.	19.05.2023	51°54′00.3″N 50°55′57.4″E	A patch of forb-fescue-feather grass steppe with a colony of steppe marmots with more than 10 living burrows, located on an almost flat area and surrounded by forest belts and agricultural fields.
2	Fig. 4	Bol’shechernigovskij Distr., 3.53 km SSW of Polyanskij Vill.	19.05.2023	51°51′28.7″N 50°52′48.5″E	A patch of forb-grass steppe with a rather large colony of steppe marmots with more than 15 living burrows, located on the slopes of the gullies and on the intergully spaces; the gully system is surrounded on all sides by agricultural fields.
3	Fig. 5	Bol’shechernigovskij Distr., 3.54 km SSW of Polyanskij Vill.	28.04.2023	51°51′27.5″N 50°52′51.2″E	Same as No 2.
4	Fig. 6	Bol’shechernigovskij Distr., 3.9 km SSW of Polyanskij Vill.	28.04.2023	51°51′09.3″N 50°53′11.6″E	A patch of forb-grass steppe with a small colony of steppe marmots on an almost flat, extended gully slope, with less than a dozen living burrows without pronounced hills of soil, which is a part of the population from localities 2 and 3.
5	Fig. 7	Pervomaiskij Distr., 6.28 km SSW of Polyanskij Vill., urochishche Pal’govo	18.05.2023	51°49′57.2″N 50°52′28.0″E	A patch of forb-fescue steppe with a small colony of steppe marmots along the bottom and lower part of the slope of a gully surrounded by agricultural fields.
6		Pervomaiskij Distr., 7.32 km SSW of Koshkin Vill., urochishche Pal’govo	19.05.2023	51°48′55.7″N 50°51′21.8″E	A patch of forb-wormwood-grass steppe with a few burrows on the slope of a gully near the top; the area is surrounded by agricultural field, except for northwest, where it borders the Talovskaya Steppe Cluster Area of the Orenburg Nature Reserve.

**Table 2.** Trophic associations.

**Dung-beetle species**	**Localities**						**Trophic associations**		
	1	2	3	4	5	6	Generalist coprophagous species	Specialist nidicolous, associated with rodents	Previous records of trophic association with *Marmota* sp.
*Aphodiusarenarius*				+				+	+
*Aphodiusbiguttatus*	+		+	+	+		+		+
*Aphodiusbimaculatus*					+		+		
*Aphodiuscitellorum*				+				+	+
*Aphodiuscoenosus*	+		+	+	+		+		+
*Aphodiusdistinctus*	+	+	+	+	+	+	+		+
*Aphodiuserraticus*			+	+	+		+		+
*Aphodiusfimetarius*				+			+		
*Aphodiusgranarius*					+		+		+
*Aphodiushaemorrhoidalis*					+		+		+
*Aphodiusimmundus*					+		+		+
*Aphodiusluridus*	+			+	+	+	+		
*Aphodiusmelanostictus*	+		+	+	+	+	+		+
*Aphodiusprodromus*						+	+		+
*Aphodiuspunctatosulcatus*					+		+		+
*Aphodiuspusillus*	+		+	+	+		+		+
*Aphodiusquadriguttatus*	+		+	+	+	+	+		+
*Aphodiusquadrimaculatus*				+	+		+		
*Aphodiussatellitus*	+			+	+		+		
*Aphodiusscrofa*				+	+		+		+
*Aphodiusvarians*					+		+		
*Caccobiusschreberi*				+			+		+
*Euoniticellusfulvus*					+		+		
*Onthophagusleucostigma*					+		+		+
*Onthophagusnuchicornis*	+		+	+		+	+		+
*Onthophagussemicornis*	+	+	+	+	+	+	+		+
*Onthophagusvacca*	+		+		+	+	+		+
*Sisyphusschaefferi*	+	+		+	+		+		+
**Total: 28**	**12**	**3**	**10**	**18**	**22**	**8**	**26**	**2**	**21**
